# Prediction of Preeclampsia Using the Soluble fms-Like Tyrosine Kinase 1 to Placental Growth Factor Ratio: A Prospective Cohort Study of Unselected Nulliparous Women

**DOI:** 10.1161/HYPERTENSIONAHA.116.08620

**Published:** 2017-03-08

**Authors:** Ulla Sovio, Francesca Gaccioli, Emma Cook, Martin Hund, D. Stephen Charnock-Jones, Gordon C.S. Smith

**Affiliations:** From the Department of Obstetrics and Gynaecology, University of Cambridge, United Kingdom (U.S., F.G., E.C., D.S.C.-J., G.C.S.S.); NIHR (National Institute for Health Research) Cambridge Comprehensive Biomedical Research Centre, United Kingdom (U.S., F.G., E.C., D.S.C.-J., G.C.S.S.); and Roche Diagnostics International, Rotkreuz, Switzerland (M.H.).

**Keywords:** clinical markers, cohort studies, immunoassay, pregnancy, risk factors

## Abstract

We sought to assess the ratio of sFlt-1 (soluble fms-like tyrosine kinase 1) to PlGF (placental growth factor) in maternal serum as a screening test for preeclampsia in unselected nulliparous women with a singleton pregnancy. We studied 4099 women recruited to the POP study (Pregnancy Outcome Prediction) (Cambridge, United Kingdom). The sFlt-1:PlGF ratio was measured using the Roche Cobas e411 platform at ≈20, ≈28, and ≈36 weeks of gestational age (wkGA). Screen positive was defined as an sFlt-1:PlGF ratio >38, but higher thresholds were also studied. At 28 wkGA, an sFlt-1:PlGF ratio >38 had a positive predictive value (PPV) of 32% for preeclampsia and preterm birth, and the PPV was similar comparing women with low and high prior risk of disease. At 36 wkGA, an sFlt-1:PlGF ratio >38 had a PPV for severe preeclampsia of 20% in high-risk women and 6.4% in low-risk women. At 36 wkGA, an sFlt-1:PlGF ratio >110 had a PPV of 30% for severe preeclampsia, and the PPV was similar comparing low- and high-risk women. Overall, at 36 wkGA, 195 (5.2%) women either had an sFlt-1:PlGF ratio of >110 or an sFlt-1:PlGF ratio >38 plus maternal risk factors: 43% of these women developed preeclampsia, about half with severe features. Among low-risk women at 36 wkGA, an sFlt-1:PlGF ratio ≤38 had a negative predictive value for severe preeclampsia of 99.2%. The sFlt-1:PlGF ratio provided clinically useful prediction of the risk of the most important manifestations of preeclampsia in a cohort of unselected nulliparous women.

## Abstract 2

Supplemental Digital Content is available in the text.

**See Editorial Commentary, pp 578–579**

Preeclampsia is one of the most common adverse outcomes of pregnancy.^1^ The condition consists of new onset hypertension and proteinuria in the second half of pregnancy but can also be superimposed on preexisting hypertension or renal disease. It is associated with increased risks of maternal and perinatal morbidity and mortality. A substantial proportion of severe adverse perinatal outcomes occur as a consequence of preterm birth because of preeclampsia, and a substantial proportion of adverse maternal outcomes occurs in severe preeclampsia.^1^

Preeclampsia is associated with an altered maternal pattern of circulating placentally derived proteins regulating angiogenesis,^2,3^ such as sFlt-1 (soluble fms-like tyrosine kinase 1) and PlGF (placental growth factor). One of the simplest methods to quantify the pattern is to calculate the ratio between these 2 angiogenic factors in maternal serum. A recent study of women with clinically suspected disease demonstrated that an sFlt-1:PlGF ratio cutoff of 38 provided clinically useful prediction of the risk of preeclampsia.^4^ Higher sFlt-1:PlGF ratios, namely >85 at 28 weeks of gestational age (wkGA) and >110 at 36 wkGA, have been shown to be more strongly associated with the risk of preeclampsia.^5^ However, evidence for the diagnostic effectiveness of the ratio in screening women without clinical suspicion of the disease is poor. A meta-analysis published in 2015 concluded that further studies were required.^6^

The aim of the present analysis was to evaluate the effectiveness of the sFlt-1:PlGF ratio as a screening test for preeclampsia in unselected nulliparous women recruited to the POP study (Pregnancy Outcome Prediction).^7,8^ Most of the participants were healthy because the cohort selection was solely based on nulliparity, singleton pregnancy, and the study catchment area. We analyzed the sFlt-1:PlGF ratio measured repeatedly at 20, 28, and 36 wkGA using the Roche Cobas e411 Elecsys immunoassay system, which has been certified by the Conformité Européenne mark for use as an in vitro medical device. Screen positive was defined on the basis of the previously described and validated cutoff of >38.^4^ We studied the most clinically important manifestations of preeclampsia, namely any severity of the disease leading to preterm birth or preeclampsia with severe features.

## Methods

### Study Design

The POP study was conducted at the Rosie Hospital, Cambridge, United Kingdom, as previously described.^7,8^ In brief, it was a prospective cohort study of nulliparous women attending the hospital for their dating ultrasound scan between January 14, 2008 and July 31, 2012 with a viable singleton pregnancy. The only clinical exclusion criterion was multiple pregnancy. The population was drawn from Cambridge and surrounding areas, with low rates of severe socioeconomic deprivation. Therefore, the cohort can be considered a population with low prior risk of disease and homogeneous from a socioeconomic perspective. Blood was obtained at the time of recruitment (not analyzed in this study). Study participants attended the NIHR (National Institute for Health Research) Cambridge Clinical Research Facility at ≈20, ≈28, and ≈36 wkGA for blood sampling and ultrasound scans. Ethical approval was given by the Cambridgeshire 2 Research Ethics Committee (reference number 07/H0308/163) and all participants provided written informed Consent.

### Outcome Data

Outcome data were ascertained by review of each woman’s paper case record by research midwives and by record linkage to clinical electronic databases of ultrasonography (Astraia, Munchen, Germany), delivery (Protos; iSoft, Banbury, United Kingdom), biochemical tests (Meditech, Westwood, MA), and neonatal intensive care (Badgernet; Clevermed Ltd, Edinburgh, United Kingdom). Where preeclampsia was suspected on the basis of these data, there was a second review of the clinical case record to confirm the diagnosis and classification (ie, with or without severe features) on the basis of the objective criteria of the 2013 ACOG Guideline (The American Congress of Obstetricians and Gynecologists; online-only Data Supplement).^9^ Superimposed preeclampsia was defined as preeclampsia in women with preexisting renal disease or hypertension. Socioeconomic status was quantified using the Index of Multiple Deprivation,^10^ and birth weight percentiles were calculated using a population-based United Kingdom reference.^11^

### Analysis Plan and Reporting

The definition of exposures, primary outcomes, secondary outcomes, and sensitivity analyses were agreed in an analysis plan (online-only Data Supplement) before performing any analysis of the sFlt-1 and PlGF data. Analyses which were not predefined are identified as such. The primary outcome for the 20 wkGA measurement was a composite of (1) preeclampsia with delivery before 28 wkGA or (2) preeclampsia with delivery before 37 wkGA where the onset of hypertension was before 28 wkGA. The primary outcome for the 28 wkGA measurement was preeclampsia with delivery before 37 wkGA. The primary outcome for the 36 wkGA measurement was preeclampsia with severe features (ie, preeclampsia with either severe hypertension or evidence of hepatic, renal, hematologic, cerebral, or pulmonary complications; online-only Data Supplement). Secondary outcomes are defined in the online-only Data Supplement. High risk of preeclampsia was defined as either (1) maternal characteristics, using the UK National Institute for Health and Care Excellence Guideline (online-only Data Supplement)^12^ or (2) elevated 20 wkGA uterine artery Doppler, defined as a mean pulsatility index in the highest decile, as previously described.^8^ Family history of preeclampsia was not included in the definition of risk status because this information was not available. The reporting of this study conforms to the STROBE (The Strengthening the Reporting of Observational Studies in Epidemiology) statement.

### Samples and Immunoassays

Serum samples were collected as previously reported^6^ and stored at −80°C. All samples used in the current analysis had not previously been thawed before the day of analysis. Researchers performing the assays were blinded to the patients’ clinical information and pregnancy outcome. Maternal serum levels of sFlt-1 and PlGF were measured using Roche Elecsys assays on the electrochemiluminescence immunoassay platform, Cobas e411 (Roche Diagnostics). Using this system, the intra-assay coefficient of variation for human serum samples is <2% for sFlt-1 and PlGF, and the interassay coefficients of variation are 2.3% to 4.3% for the sFlt-1 assay and 2.7% to 4.1% for the PlGF assay. Screen positive was defined as sFlt-1:PlGF ratio of >38.^4^ We also studied more severe elevation of the ratio, namely, >85 at 28 wkGA and >110 at 36 wkGA.^5^

### Statistical Analysis

Full details of the statistical analysis are described in the Analysis plan (online-only Data Supplement). In brief, standard screening summary statistics were calculated from 2×2 tables. In addition, sFlt-1:PlGF ratio was analyzed as a continuous variable using the area under the receiver operating characteristic curve (AUROCC). Time-to-event analysis was performed where delivery with the given outcome was the event and delivery without the outcome was treated as a competing risk, and this method was used to generate plots of the cumulative incidence of the outcome from the time of measurement. All analyses were performed using Stata 14.1.

### Exclusions and Missing Data

Of the 4512 women recruited, 67 (1.5%) women withdrew and 233 (5.2%) delivered elsewhere, leaving 4212 eligible women.^8^ Of these, 5 (0.1%) did not have preeclampsia status available, and 108 (2.6%) did not have any sFlt-1:PlGF measurements available from the 28 or 36 wkGA visits.

## Results

### Description of the Study Cohort

The study group consisted of 4099 women, of whom 3953 had sFlt-1:PlGF measurement available from the 20 wkGA visit, 3989 from the 28 wkGA visit, and 3776 from the 36 wkGA visit. The overall incidence of preeclampsia was 6.5% (265/4099). The incidence of preterm preeclampsia with onset before 28 wkGA after the 20 wkGA measurement was 0.10% (4/3953). The incidence of preeclampsia leading to preterm delivery following the 28 wkGA measurement was 0.65% (26/3989). The incidence of delivery with severe preeclampsia after the 36 wkGA measurement was 2.8% (106/3776). The characteristics of the cohort are tabulated according to their experience of hypertensive complications of pregnancy (Table 1). In normotensive women, the median sFlt-1:PlGF ratios were 6.5, 2.7, and 11.2 at 20, 28, and 36 wkGA, respectively. The median ratio at 28 wkGA was 6.5 (interquartile range, 3.4–22.5) in women who developed preterm preeclampsia, and the median ratio at 36 wkGA was 42.3 (interquartile range, 22.6–88.2) in women who had severe preeclampsia.

**Table 1.**
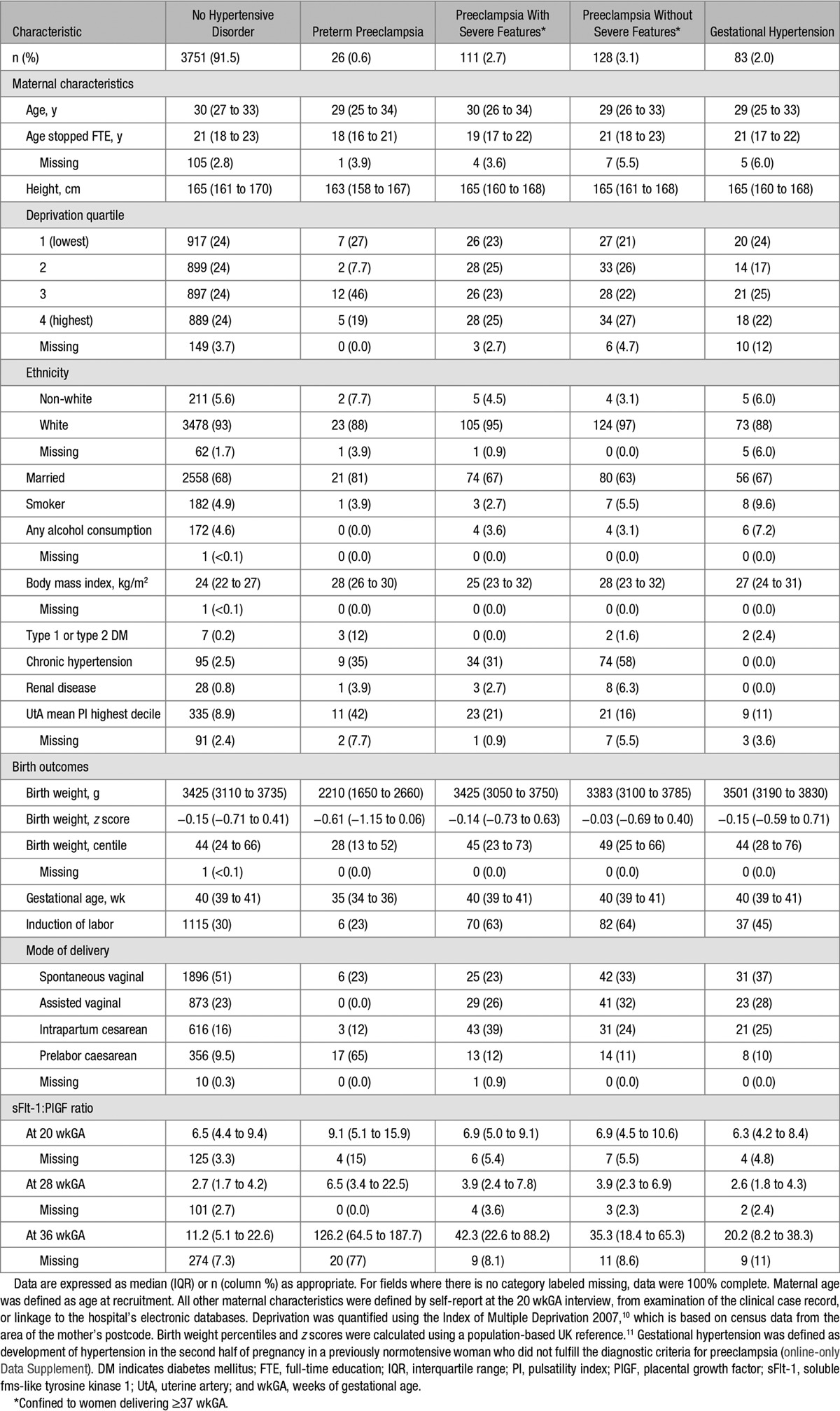
Characteristics of the Study Cohort (n=4099)

### Screening Performance at 20 wkGA

**Figure 1.**
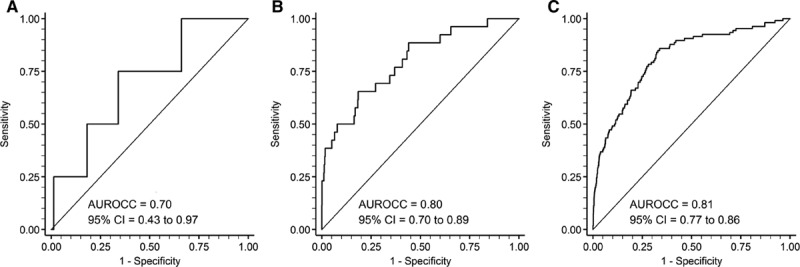
Receiver operating characteristic curve analysis of the relationship between (**A**) sFlt-1 (soluble fms-like tyrosine kinase 1) to PlGF (placental growth factor) ratio at 20 weeks of gestational age (wkGA) and (1) preeclampsia with delivery before 28 wkGA or (2) preeclampsia with delivery before 37 weeks where the onset of hypertension was before 28 wkGA (n=4), (**B**) sFlt-1:PlGF at 28 wkGA and preeclampsia leading to preterm birth (n=26), and (**C**) sFlt-1:PlGF at 36 wkGA and severe preeclampsia (n=106). The continuous sFlt-1:PlGF ratio is used, and the area under the receiver operating characteristic curve (AUROCC) with 95% confidence interval (CI) is given for each analysis.

None of the 4 women who experienced the primary outcome after the 20 wkGA measurement had an sFlt-1:PlGF ratio >38. The AUROCC for the sFlt-1:PlGF ratio was 0.70 (95% confidence interval, 0.43–0.97; Figure 1A). Ten women had a ratio >38, and 1 woman had a ratio >85 at 20 wkGA. Further analysis was not performed because of the small number of events.

### Screening Performance at 28 wkGA

The AUROCC for the sFlt-1:PlGF ratio was 0.80 (95% confidence interval, 0.70–0.89; Figure 1B). Women with an sFlt-1:PlGF ratio >38 (n=19) had an incidence of preeclampsia leading to preterm delivery of 32% (Table 2). The positive predictive value (PPV) was similar in low- and high-risk women (33% versus 31%, respectively; *P*=0.91).

**Table 2.**
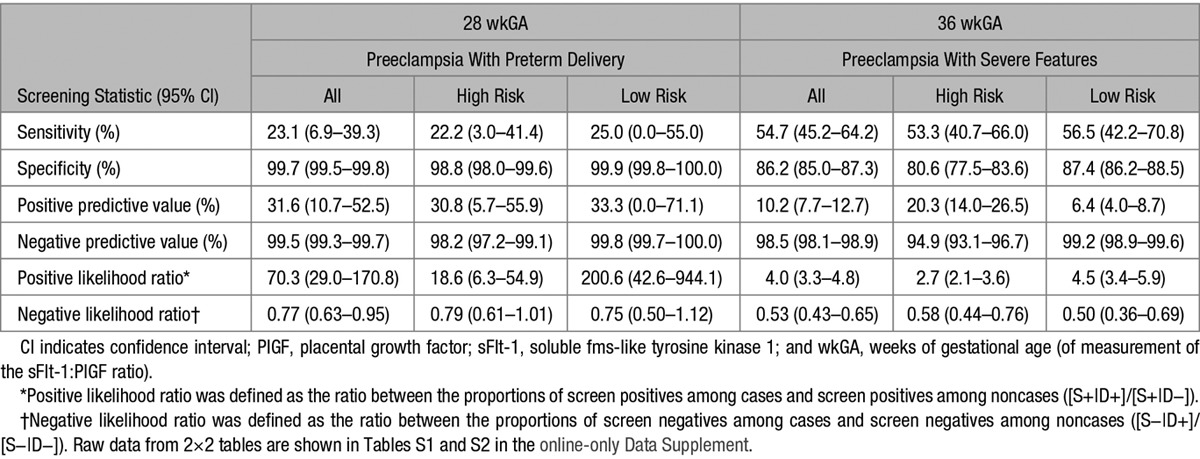
Screening Statistics for the Primary Outcomes by Maternal Risk Status Using the Threshold of sFlt-1:PlGF Ratio of >38 at 28 and 36 wkGA

### Screening Performance at 36 wkGA

The AUROCC for the sFlt-1:PlGF ratio was 0.81 (95% confidence interval, 0.77–0.86; Figure 1C). Women with an sFlt-1:PlGF ratio >38 (n=566) had an incidence of severe preeclampsia of 10% (Table 2). The PPV was 20% in high-risk women and 6.4% in low-risk women. Among women with no prior risk factors, an sFlt-1:PlGF ratio ≤38 had a high negative predictive value for subsequent development of severe preeclampsia (>99%).

### Analysis of Severe Elevation of the sFlt-1:PlGF Ratio

We studied more severe elevation of the ratio, using predefined thresholds, namely, 85 at 28 wkGA and 110 at 36 wkGA (Table 3). Only 7 women had an sFlt-1:PlGF ratio >85 at 28 wkGA. However, 4 out of 7 delivered preterm with a diagnosis of preeclampsia (PPV=57%). At 36 wkGA, 70 women had an sFlt-1:PlGF ratio >110, and 21 developed severe preeclampsia (PPV=30%). The PPV was similar comparing women with and without prior risk factors (36% and 24%, respectively).

**Table 3.**
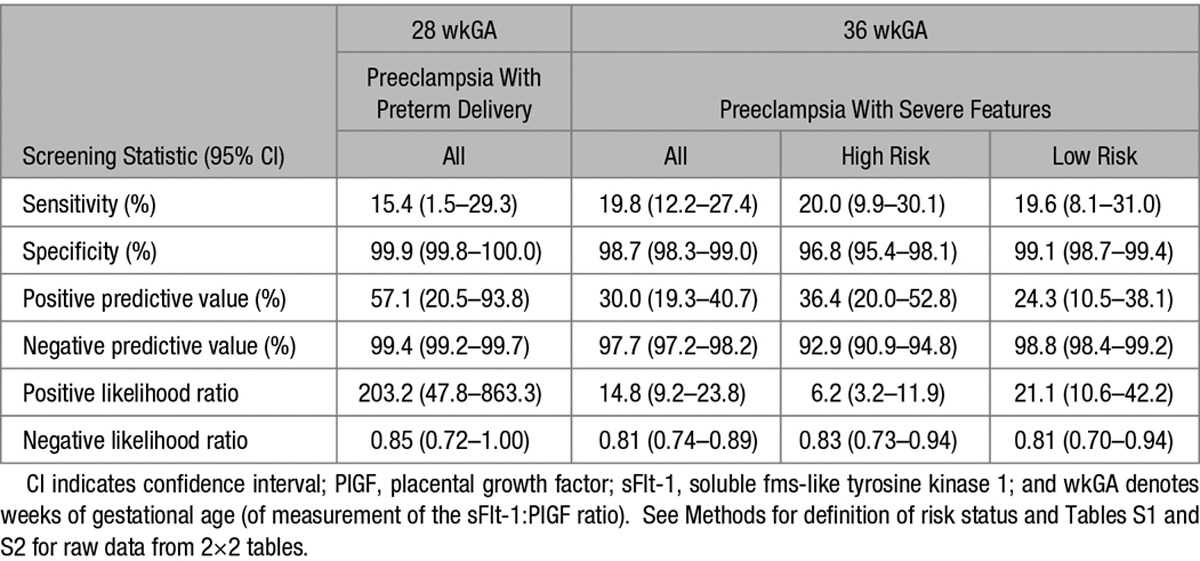
Screening Statistics for the Primary Outcomes by Maternal Risk Status Using the Threshold of sFlt-1:PlGF Ratio of >85 at 28 wkGA and >110 at 36 wkGA

### Screening Performance at 36 wkGA Using the sFlt-1:PlGF Ratio Combined With Maternal Risk Factors

We then analyzed a composite definition of screen positive at 36 wkGA, namely, an sFlt-1:PlGF ratio of >110 irrespective of maternal risk factors or an sFlt-1:PlGF ratio >38 combined with maternal risk factors. A total of 195 (5.2%) women screened positive by this definition and 43% of them subsequently delivered with a diagnosis of preeclampsia: 41 women (PPV=21%) developed preeclampsia with severe features, and 43 (PPV=22%) developed preeclampsia without severe features. The characteristics and outcomes for the 195 women are summarized (online-only Data Supplement).

### Time-to-Event Analysis

**Figure 2.**
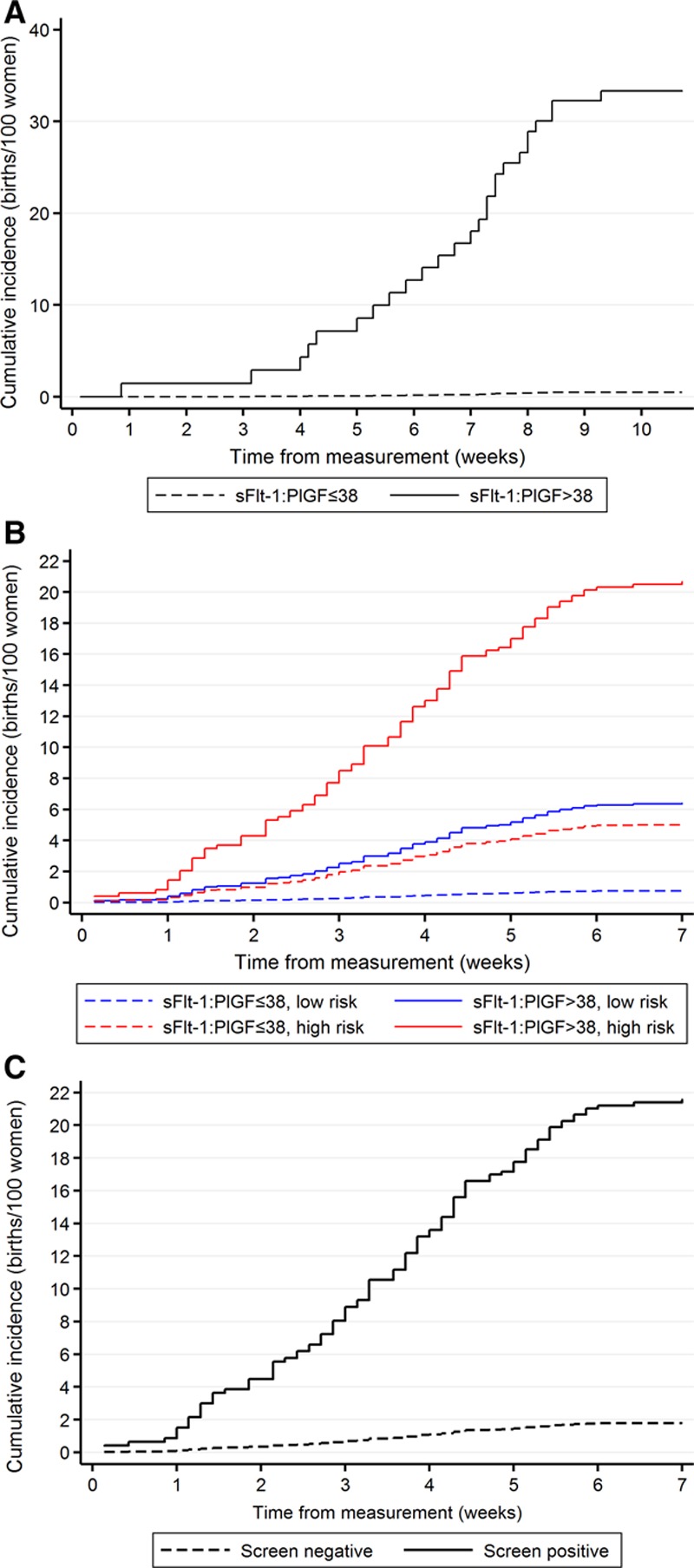
Cumulative incidence of the primary outcomes (Methods) by sFlt-1 (soluble fms-like tyrosine kinase 1) to PlGF (placental growth factor) ratio: (**A**) sFlt-1:PlGF at 28 wkGA and preeclampsia leading to preterm birth, (**B**) sFlt-1:PlGF at 36 wkGA and severe preeclampsia, stratified by maternal risk. High risk was defined on the basis of maternal risk factors or 20 wkGA uterine artery Doppler (see Methods for details), and (**C**) composite risk status at 36 wkGA. Screen positive was defined as (1) sFlt-1:PlGF ratio of >38 and maternal risk factors or (2) sFlt-1:PlGF ratio >110 irrespective of maternal risk factors. Screen negative was defined as all other women. Delivery without the given primary outcome was treated as a competing risk in all 3 analyses. Hence, the maximum value of the cumulative incidence is the same as the positive predictive value, and the curve illustrates the distribution of the timing of the deliveries with the outcome in question.

We plotted the cumulative incidence of the primary outcomes after the 28 and 36 wkGA measurements of the sFlt-1:PlGF ratio using a competing risks model (Figure 2A and B). The 36 wkGA measurements were stratified by maternal risk status (Figure 2B). In both cases, the curves started to deviate at least 1 week after the time of measurement, and the proportional increase in risk was maintained over the 7 to 8 weeks after the test. We also plotted the cumulative incidence of preeclampsia for women at 36 wkGA with the composite definition of screen positive (Figure 2C), that is, a ratio >38 plus risk factors or a ratio of >110 irrespective of risk factors. Delivery without the primary outcome was treated as a competing risk in all 3 analyses. In all 3 plots, it is evident that >90% of the deliveries in the highest risk group occurred >1 week from the time of measurement of the ratio.

## Discussion

The main finding of this study is that, in a cohort of unselected, first singleton pregnancies, measurement of the sFlt-1:PlGF ratio identified women with a high absolute risk of experiencing the clinically most important manifestations of preeclampsia. At 28 wkGA, an sFlt-1:PlGF ratio >38 identified women with a high risk (>30%) of subsequently delivering preterm with preeclampsia. Women who had a more severe elevation of the ratio (>85) had nearly 60% risk of delivering preterm with preeclampsia, whereas >99% of women who had a ratio of <38 did not develop the outcome. At 36 wkGA, ≈5% of women were identified as high risk on the basis of either an sFlt-1:PlGF ratio of >110 or an sFlt-1:PlGF ratio >38 plus maternal risk factors. Of this group, 43% were subsequently delivered with a diagnosis of preeclampsia, and about half of these cases were severe. Approximately 70% of women were identified as low risk at 36 wkGA, that is, they had no maternal risk factors and an sFlt-1:PlGF ratio ≤38; their risk of developing severe preeclampsia was <1%.

Screening is generally only conducted when there are evidence-based interventions which mitigate the risk. A key element in the study design was to make a measurement close to term (36 wkGA), the rationale being that delivery is the main intervention to prevent preeclampsia. This can more easily and more safely be performed at term.^13^ Moreover, a randomized controlled trial has shown improved outcome after immediate induction of labor compared with expectant management in women with gestational hypertension or mild preeclampsia near term.^14^ In this study, we evaluated the diagnostic effectiveness of the sFlt-1:PlGF ratio in identifying women at risk of developing preeclampsia. In light of our results, we hypothesize that one approach to reducing the burden of morbidity associated with preeclampsia could be to screen nulliparous women at 36 wkGA using maternal risk factors and sFlt-1:PlGF ratio, monitor screen positive women closely, and perform induction of labor before the development of severe disease. This hypothesis could be readily tested in a randomized controlled trial evaluating whether the introduction of the screening test improves pregnancy outcome (clinical effectiveness). Such an intervention is unlikely to cause harm, and we have recently demonstrated that routine induction of labor at 39 wkGA does not increase the risk of cesarean delivery or perinatal morbidity in another high-risk population of nulliparous women, namely, those aged ≥35.^15^

The sFlt-1:PlGF ratio was also informative for the risk of preterm preeclampsia. Women with a ratio >38 at 28 wkGA had a 32% risk of preterm delivery with preeclampsia, and the ratio >38 had a high positive likelihood ratio (≈70). This may reflect the fact that this threshold represents much more significant elevation at 28 wkGA (99.5th percentile) than 36 wkGA (85th percentile). However, the sensitivity of the sFlt-1:PlGF ratio >38 at 28 wkGA was only 23%. Although the POP study included mostly healthy women, this is consistent with the findings in women clinically suspected to have preeclampsia, where an elevated sFlt-1:PlGF ratio between 24 and 37 wkGA was also associated with an increased risk of developing the disorder within 4 weeks after the measurement.^16^ Hence, although the test provides clinically useful prediction of risk for a small proportion of women, the majority of women experiencing the disease would not be identified using the test at 28 wkGA. However, the AUROCC for the sFlt-1:PlGF ratio at 28 wkGA for preterm preeclampsia was 0.80. It is possible that combining the ratio with other measurements (clinical, biomarker, and ultrasonic) in a multivariable model might provide better risk prediction. This is an area of further investigation, which will be hopefully paralleled by studies aiming at the implementation of better treatment options. Currently, the main limitation of the clinical usefulness of the 28 wkGA measurement is the lack of a clearly effective intervention mitigating the risks for those who screen positive, other than close monitoring of the patient. Another important area for further study is to refine the estimation of risk in women whose 36 wkGA assessment identified them as being at intermediate risk (5%–6%) of severe preeclampsia, namely, women with an sFlt-1:PlGF ratio >38 and no risk factors or risk factors but a ratio of ≤38. Possible approaches include identifying other informative biomarkers or repeating measurement of the sFlt-1:PlGF ratio after 36 wkGA.

This study had many methodological strengths over previous studies. First, the size was sufficiently large that we were able to study the variants of preeclampsia associated with the most severe complications. Second, we used a clinically validated assay where the definition of screen positive was based on prior studies which had identified and validated the chosen threshold. Moreover, the sFlt-1:PlGF ratio can be calculated without modeling in relation to gestational age or maternal characteristics, and in this study, clinical care was provided without knowledge of the test result. Finally, the analyses in this study were planned and specified in advance.

A large-scale study on screening women using the sFlt-1:PlGF ratio has recently been reported.^17^ However, their findings are difficult to compare to this study because they pooled the results from a wide range of gestational ages (30–37 wkGA). Moreover, almost half of their population consisted of multiparous women who had not previously experienced preeclampsia, and this is a group with a very low prior risk of disease. The PPV of a test depends both on the a priori risk and the positive likelihood ratio. It is difficult to interpret a summary estimate of PPV when almost half the population has a very low prior risk of the outcome. Another large study based on a multiethnic cohort of nulliparous women and high-risk parous women concluded that angiogenic biomarkers measured in the first half of pregnancy performed poorly for predicting later development of preeclampsia.^18^ They also observed, however, that the measurements became more strongly predictive when made closer to disease onset, but the analyses of late pregnancy data were limited by high rates of missing biomarker data (20%–30%). The focus of this study on nulliparous women was purposeful. One of the strongest clinical predictors of the risk of preeclampsia is whether a woman has a previous history of pregnancy affected by the condition. This information dominates risk prediction in multiparous women but is necessarily absent among nulliparous women. Another purposeful feature of the design of this study was measurement of biomarkers throughout gestation. Results reported in the current and previous studies^13^ suggest that screening tests for pregnancy complications have a better predictive value when performed close to disease onset.

Many studies have evaluated trying to predict preeclampsia solely using measurements made in the first trimester. Statistical models are used to determine prior risk. Further modeling is used to process values of first trimester uterine artery Doppler flow velocimetry and to convert first trimester protein concentrations into gestational age-corrected multiples of the median. Two models were externally evaluated in a prospective cohort study in Norway, where measurements were performed between 11 and 14 wkGA.^19^ A study-derived threshold (10% false-positive rate) yielded PPVs of 5% to 12%, a sensitivity of 40% and positive likelihood ratios of 1.5 to 3.6 for all preeclampsia cases. Although the nature of that study does not allow direct comparison with this analysis, the current approach may be more likely to be clinically applicable, given that (1) the definition of screen positive was externally defined, (2) the PPVs were higher, (3) the outcome was confined to the clinically most significant cases, and (4) the handling of clinical and biochemical predictors is simpler.

## Perspectives

We conclude that measurement of the sFlt-1:PlGF ratio at 36 wkGA, combined with maternal risk factors, provides clinically useful prediction of the risk of preeclampsia at term for ≈3 quarters of unselected nulliparous women, identifying 5% of them as high risk and 70% as low risk. Screening the pregnant nulliparous population in late pregnancy using this measurement could plausibly improve maternal and perinatal outcome when coupled with close monitoring and induction of labor, and this would be an appropriate focus for future randomized controlled trials. Women with extreme elevation of the ratio at 28 wkGA have high absolute risks of preterm disease, and the test may be useful to identify women for the evaluation of candidate disease-modifying therapies as they become available.

## Acknowledgments

We are extremely grateful to the participants in the POP study (Pregnancy Outcome Prediction). We are grateful to Leah Bibby, Samudra Ranawaka, Katrina Holmes, Josephine Gill, and Ryan Millar for their technical assistance in performing the biochemical assays, and to Drs Rabia Zill-e-Huma and Amr Gebril for reviewing the clinical case records.

## Sources of Funding

The work was supported by the National Institute for Health Research (NIHR) Cambridge Comprehensive Biomedical Research Centre (Women’s Health theme), and project grants from the Medical Research Council (United Kingdom; G1100221) and the Stillbirth and neonatal death society (Sands). The study was also supported by Roche Diagnostics (provision of equipment and reagents for analysis of sFlt-1 [soluble fms-like tyrosine kinase 1] and PlGF [placental growth factor]), by GE Healthcare (donation of 2 Voluson i ultrasound systems for this study), and by the NIHR Cambridge Clinical Research Facility, where all research visits took place.

## Disclosures

M. Hund reports being an employee of Roche Diagnostics, holding stock in Roche, having a pending patent related to the sFlt-1 (soluble fms-like tyrosine kinase 1) to PlGF (placental growth factor) or endoglin:PlGF ratio to rule out onset of preeclampsia in pregnant women within a certain time period (PCT/EP2013/063115), holding pending patents related to the dynamic of sFlt-1 or endoglin:PlGF ratio as an indicator for imminent preeclampsia or the HELLP (hemolysis, elevated liver enzymes, low platelet count) syndrome or both (PCT/EP2012/072157) and the prediction of postpartum HELLP syndrome, postpartum eclampsia, or postpartum preeclampsia (PCT/EP2015/051457). G.C.S. Smith reports equipment loans and consumable support from Roche Diagnostics. The other authors report no conflicts.

## Supplementary Material

## Novelty and Significance

### What Is New?

Among a population of nulliparous women at mixed risk of disease:

- An sFlt-1 (soluble fms-like tyrosine kinase 1) to PlGF (placental growth factor) ratio >38 at 28 weeks of gestational age identified women with a high risk (>30%) of subsequently delivering preterm with preeclampsia.
- An sFlt-1:PlGF ratio >110 at 36 weeks of gestational age identified women with a high risk (>30%) of subsequently experiencing severe preeclampsia.
- An sFlt-1:PlGF ratio between >38 and <110 at 36 weeks of gestational age was only associated with a high absolute risk (>20%) of subsequently experiencing severe preeclampsia if the mother had additional risk factors.

### What Is Relevant?

- Measurement of the sFlt-1:PlGF ratio provides clinically useful information on risk of the most clinically important manifestations of preeclampsia among women having first pregnancies.
- This identifies potential new approaches to trials of screening and intervention.

### Summary

Very high elevation of the sFlt-1:PlGF ratio identifies women with high absolute risks of preterm or severe preeclampsia, and moderate elevation is informative of risk when combined with maternal risk factors.
