# Supplementary material for: Prediction of Preeclampsia Using the Soluble fms-Like Tyrosine Kinase 1 to Placental Growth Factor Ratio: A Prospective Cohort Study of Unselected Nulliparous Women
Source: Hypertension. 2017 Mar 8;69(4):731–8. doi: 10.1161/HYPERTENSIONAHA.116.08620 (PMC5344177; doi:10.1161/HYPERTENSIONAHA.116.08620)
Supplement: Supplementary file 1 [file hyp-69-731-s001.docx]

**ONLINE SUPPLEMENTAL MATERIAL**

**PREDICTION OF PREECLAMPSIA USING THE sFlt-1:PlGF RATIO: A PROSPECTIVE COHORT STUDY OF UNSELECTED NULLIPAROUS WOMEN**

Ulla Sovio, Ph.D.^a,1,2^; Francesca Gaccioli, Ph.D.^a,1^; Emma Cook, B.Sc.^a^; Martin Hund, Ph.D.^b^; D. Stephen Charnock-Jones, Ph.D.^a^; Gordon C. S. Smith, D.Sc.^a^

^a^Department of Obstetrics and Gynaecology, University of Cambridge; NIHR Cambridge Comprehensive Biomedical Research Centre, Cambridge, UK.

^b^Roche Diagnostics International, Rotkreuz, Switzerland.

**Supplemental Methods**

*Diagnosis of preeclampsia (based on the 2013 ACOG Guideline* (PMID 24150027)*)*

Hypertension, for the purposes of diagnosis of preeclampsia, was defined as one or more of the following:

- 2x diastolic blood pressure (DBP) >89 mmHg at least 4 hours apart
- 1x DBP >89 mmHg + (new antihypertensive treatment AND/OR magnesium sulphate)
- 1x DBP>109 mmHg
- 2x systolic blood pressure (SBP) >139 mmHg at least 4 hours apart
- 1x SBP >139 mmHg + (new antihypertensive treatment AND/OR magnesium sulphate)
- 1x SBP >159 mmHg

Severe hypertension, for the purposes of diagnosis of preeclampsia with severe features, was defined as any one of the following:

- 2x DBP >109 mmHg at least 4 hours apart
- 2x SBP >159 mmHg at least 4 hours apart
- 1x DBP >109 mmHg + (new antihypertensive Rx AND/OR magnesium sulphate)
- 1x SBP >159 mmHg + (new antihypertensive Rx AND/OR magnesium sulphate)

Proteinuria was defined as one or more of the following:

- Greater than or equal to 300 mg per L in a 24 hour urine collection
- 2x dipstick reading of 1+ or greater 4 hours apart at >20 weeks gestational age

Low platelets was defined as:

- Platelets <100 at ≥ 24 weeks gestational age up to 48h after delivery in a woman with normal levels (>150) <20 weeks

Elevated creatinine was defined as:

- Creatinine >99mmol/L at ≥ 24 weeks gestational age up to 48h after delivery in women with normal levels or unrecorded <20 weeks. If the first onset of the hypertension was after delivery measurements up to 7d after birth were included in the definition.

Elevated alanine transaminase (ALT) was defined as:

- ALT >49 at ≥ 24 weeks gestational age up to 48h after delivery in a woman with normal levels or unrecorded <20 weeks

Pulmonary oedema was defined on the basis of whether it was documented as being present when reviewing the paper case record

Severe cerebral or visual disturbance was defined as:

- Severe cerebral or visual disturbance documented in the paper case record, plus evidence of significant clinical concern, including 2 or more measures of uric acid

Preeclampsia was defined as:

- Hypertension (defined above) AND (Proteinuria AND/OR low platelets AND/OR elevated ALT AND/OR elevated creatinine AND/OR pulmonary oedema AND/OR severe cerebral/visual symptoms)

Preeclampsia with severe features was defined as:

- Preeclampsia AND (severe hypertension AND/OR low platelets AND/OR elevated ALT AND/OR elevated creatinine AND/OR pulmonary oedema AND/OR severe cerebral/visual symptoms)

Gestational hypertension (GH) was defined as hypertension (as above) but not preeclampsia in a woman who was not hypertensive prior to 20 weeks and had no history of renal disease or essential hypertension.

Severe gestational hypertension was defined as GH AND severe hypertension

Pre-existing hypertension was defined as:

- Record of any documented history of essential hypertension or documented past or present use of anti-hypertensives when booking for antenatal care AND/OR any DBP>89 prior to 20 weeks gestational age AND/OR any SBP>139 prior to 20 weeks gestational age

Pre-existing renal disease was defined as:

- Record of any documented history of renal disease AND/OR heavy proteinuria prior to 20 weeks

Super-imposed preeclampsia

- If there is pre-existing hypertension or renal disease, then preeclampsia is defined as super-imposed. Diagnosis of superimposed preeclampsia required either new proteinuria or evidence of systemic involvement.

*Analysis plan*

**Analysis plan for assessing the diagnostic effectiveness of the sFlt-1/PlGF ratio as a screening test for pre-eclampsia in the Pregnancy Outcome Prediction study (PMID 19019223 & 26360240).**

1.1 Defining exposure

An existing study, PROGNOSIS (Hund et al, PMID 25230734), has evaluated the predictive value of knowing the Elecsys sFlt-1/PlGF immunoassay ratio in women with suspected pre-eclampsia (Zeisler et al, PMID 26735990). In that study, the ratio was treated as elevated when the value was >38 (ruling in preeclampsia within 4 weeks). We will examine the relationship between measurements above and below this threshold and the risk of pre-eclampsia using blood samples collected from a prospective cohort of nulliparous women, conducted in Cambridge, UK, namely, the Pregnancy Outcome Prediction study (PMID 19019223 & 26360240). The analysis will focus on three time points of measurement of the sFlt-1/PlGF ratio: (i) at ~20 weeks, (ii) at ~28 weeks, and (iii) at ~36 weeks. We will also evaluate the screening properties of greater elevation of the ratio (>85 at 20 & 28 weeks and >110 at 36 weeks) which have been previously described as diagnostic for pre-eclampsia (Stepan et al, PMID 25736847).

1.2 Defining outcome

All definitions of outcome will be as per the 2013 ACOG classification (PMID 24150027). Super-imposed pre-eclampsia is defined as pre-eclampsia in women with previous renal disease or hypertension. "Severe" is defined in the ACOG Guideline.

1.3 Primary and secondary outcomes

*1.3.1 The sFlt-1/PlGF ratio at ~20 weeks*

The primary outcome for the 20 week measurement is pre-eclampsia with delivery prior to 28 weeks or pre-eclampsia with delivery prior to 37 weeks and where the onset of hypertension was prior to 28 weeks.

*1.3.2 The sFlt-1/PlGF ratio at ~28 weeks*

The primary outcome for the 28 week measurement is pre-eclampsia with delivery prior to 37 weeks. Secondary outcomes for the 28 week measurement are (a) pre-eclampsia, with onset of hypertension prior to 37 weeks, (b) gestational hypertension, with delivery prior to 37 weeks, (c) gestational hypertension, with onset of hypertension prior to 37 weeks, (d) either pre-eclampsia or gestational hypertension with onset of hypertension prior to 37 weeks.

*1.3.3 The sFlt-1/PlGF ratio at ~36 weeks*

The primary outcome for the 36 week measurement is pre-eclampsia with severe features. Secondary outcomes for the 36 week measurement are (a) pre-eclampsia without severe features, (b) severe gestational hypertension, (c) gestational hypertension, (d) either pre-eclampsia or gestational hypertension.

1.3.4 Sensitivity analyses

1.3.4.1 We will compare associations between super-imposed pre-eclampsia and pre-eclampsia in women without prior hypertension or renal disease.

1.3.4.2 We will also compare the predictive ability of the test, stratifying the cohort on the basis of being at high risk of pre-eclampsia. This will be defined on the basis of maternal characteristics (using the NICE Guideline recommendation of 2 moderate or 1 high risk factor) or the 20 week uterine artery Doppler (defined as a mean pulsatility index >90^th^ percentile).

1.3.4.3 At each time point (20, 28 or 36 weeks), we will also analyse associations based on the previous measurement (i.e. 12, 20 or 28 weeks, respectively). We will then determine whether the associations between the outcomes and the sFlt-1/PlGF ratio differ according to the trend of increasing or decreasing value. Low will be defined as <38, moderate as 38-85/110 (depending on GA), and high as >85 at 20 or 28 weeks, or >110 at 36 weeks.

1.4 Analytic approach

Analysis will be performed using 2x2 tables and calculation of standard screening summary statistics (sensitivity, specificity, positive predictive value, negative predictive value, and positive and negative likelihood ratios). We will also describe the association between pre-eclampsia and the sFlt-1/PlGF ratio as a continuous variable by plotting the receiver operating characteristic (ROC) curve and estimating the c statistic (= the area under the ROC curve). We will perform analyses for a ratio of >38 ruling in pre-eclampsia, and a ratio of <38 for ruling out pre-eclampsia. We will repeat the analysis for severe elevation of the ratio (>85 at 20 or 28 weeks, or >110 at 36 weeks).

Finally, we will analyse by time to event methods, i.e. a Kaplan-Meier plot of cumulative incidence of pre-eclampsia from time of measurement, and use a Cox model to generate HR for subsequent diagnosis with pre-eclampsia. We may also use time to event methods that account for competing risks (Akolekar et al, 2013, PMID 22906914). We will use these methods to perform secondary analyses of screening performance to rule in/out pre-eclampsia for 1, 2, 4 and 6 weeks.

1.5 Exploratory analyses

We will compare the predictive ability of the ratio using ROC curve analyses for different phenotypes of pre-eclampsia, specifically, associated with (i) maternal renal dysfunction, (ii) HELLP syndrome, or (iii) fetal growth restriction.

We will also determine the relationship between the risk of pre-eclampsia and the trend of the level of the sFlt-1/PlGF ratio (i.e. increasing or decreasing) treating the ratio as a continuous variable and modelling the change across gestation.

1.6 Presentation of results.

In the publication, primary and secondary outcomes will be defined as above. Any analyses not described above will be clearly identified as not part of the original analysis plan.

1.7 Statement

The above plan was drawn up prior to any analysis of the relationship between sFlt-1, PlGF, or the ratio of sFlt-1/PlGF and the maternal risk of pre-eclampsia in the Pregnancy Outcome Prediction study.

*Missing data*

Of 4,512 women recruited to the study, we excluded 300 (6.6%) women who were not eligible, 5 (0.1%) who did not have preeclampsia status available and 108 (2.6%) who did not have any sFlt-1:PlGF measurements available from the 28 or 36 week visits.

*Trend analyses*

Change in the sFlt-1:PlGF ratio was defined as the change in the measurement between the 28 and 36 week visits divided by the time difference between the measurements in weeks. The association between the change in the sFlt-1:PlGF ratio was quantified using the c statistic. We then determined whether the associations between the primary outcome and the sFlt-1:PlGF ratio differed according to the trend of increasing value, which was analysed in tertiles to ensure an adequate number of cases in each stratum.

**Supplemental Results**

*Missing data*

Among the 4,099 women included in the study population, 3,953 had sFlt-1:PlGF measurement available from the 20 week visit, 3,989 from the 28 week visit and 3,776 from the 36 week visit. Among the variables used for defining risk status based on maternal characteristics, the proportion of missing values was low (105/4,099 = 2.6% in total, including 104 missing values in UtA PI from the 20 week visit and one missing value in BMI from the 12 week visit). We replaced them with the most common value for the purpose of defining high risk of preeclampsia in the entire study population, i.e. UtA PI ≤90^th^ percentile and BMI<35 kg/m^2^.

*Descriptive statistics*

Overall, 81% of the women were classified into the low risk and 19% were classified into the high risk category, based on maternal characteristics without considering the sFlt-1:PlGF measurement. Raw data on screening for the primary outcomes by maternal risk status using different thresholds is given in **Tables S1 and S2**.

A total of 195 women were screen positive using a composite definition at 36wkGA (sFlt-1:PlGF ratio >110 irrespective of maternal risk factors or sFlt-1:PlGF ratio >38 with maternal risk factors). Their characteristics and outcomes are summarized in **Table S3**.

In total, 21 cases of severe preeclampsia had sFlt-1:PlGF ratio >110 at 36wkGA, and 32 cases had sFlt-1:PlGF ratio >38 and maternal risk factors. Twelve of these overlap (20 women had maternal risk factors and sFlt-1/PlGF ratio between 38 and 110, and 9 women had sFlt-1/PlGF ratio >110 but no maternal risk factors).

A total of 2,660 women were screen negative using a definition of sFlt-1:PlGF ratio of ≤38 and the absence of maternal risk factors. Their characteristics and outcomes are summarized in **Table S4**.

Of the 106 women who had pre-eclampsia with severe features, 72 had proteinuria, 69 had abnormal blood results (including 43 elevated creatinine, 30 elevated ALT and 18 low platelets), 67 had severe hypertension, ten had severe cerebral or visual disturbances and two had pulmonary oedema. The overlap of five of these features including blood abnormalities, hypertension and severe cerebral/visual disturbances has been described using a Venn diagram (**Figure S1**).

*Model performance*

For the 28wkGA and 36wkGA measurements, the area under the ROC curve (AUROCC) was similar regardless of the maternal risk status (**Figures S2 and S3**). The model performance was generally weaker for the secondary outcomes (**Figures S4 and S5**). When the primary outcomes were compared according to whether the preeclampsia was superimposed on prior hypertension or renal disease, the AUROCC was ~0.8 in all analyses (**Figures S6 and S7**). No further analysis was performed using the 20wkGA measurement due to small number of events.

Of the secondary outcomes, gestational hypertension leading to preterm birth was strongly associated with the continuous sFlt-1:PlGF ratio at 28wkGA (**Table S5**). The association between the ratio measured at 36wkGA and any gestational hypertension was much weaker (AUROCC = 0.88 vs. 0.62, respectively).

Finally, the associations were analysed by phenotype of preeclampsia. The number of cases was small in the analysis of the sFlt-1:PlGF ratio and preeclampsia with renal dysfunction (n=1) or HELLP syndrome (n=2) leading to preterm birth (**Figure S8**). The sFlt-1:PlGF ratio at 36wkGA was strongly associated with preeclampsia combined with HELLP syndrome but not with preeclampsia combined with renal dysfunction (**Figure S9**).

*Trend analyses*

The primary outcome at 36wkGA was additionally analysed in relation to change in the sFlt-1:PlGF ratio between 28 and 36 week visit. A total of 3,666 women had both measurements available. The median change was 1.11 (IQR 0.35-2.67) per wkGA. The change in the sFlt-1:PlGF ratio was highly correlated with the sFlt-1:PlGF ratio at 36wkGA (spearman correlation coefficient = 0.98). The variation in the ratio at 36wkGA was much larger than at 28wkGA, and the change in the ratio was driven by the 36wkGA measurement. Therefore, its association with the primary outcome at 36wkGA was very similar: the c statistic was 0.82 (95% CI 0.77–0.86). We did not observe substantial differences in the associations between the primary outcome and the sFlt-1:PlGF ratio according to the trend of increasing value although the c statistic was slightly higher in the highest tertile (**Table S6**).

*Proximity of measurement and the risk of preeclampsia*

We performed an analysis not described in the analysis plan to determine the relationship between the timing of screening and the strength of association with preeclampsia. In order to allow comparison of measurements made at different gestational ages, we studied the top decile of sFlt-1:PlGF ratio using the distribution of values observed in this study. The risk of preeclampsia resulting in preterm birth was increased in women with values of the ratio in the top decile at both 20wkGA and 28wkGA (**Figure S10**). However, the association was stronger for measurement at 28wkGA. Top decile of the sFlt-1:PlGF ratio at 20wkGA was not associated with the risk of preeclampsia with severe features at or near term but there were associations with both the 28wkGA and 36wkGA measurements. However, the association was stronger at 36wkGA. Hence, the ratio was most strongly predictive when it was measured closest to the timing of the given event.

*Cut offs within the study cohort*

We generated data derived cut offs for the prediction of preeclampsia based on the ROC curve using the sFlt-1:PlGF ratio at 28 weeks (Figure 1B). The cut off of sFlt-1:PlGF ratio of 14.6 resulted in 38% sensitivity and 98% specificity. We repeated the primary analysis with the new cut offs (**Table S7**). The positive predictive values were 12.7% for all women and 20.6% and 6.7% for high risk and low risk women, respectively.

**Table S1.** Raw data on screening for the primary outcomes by maternal risk status using the threshold of sFlt-1:PlGF ratio of >38 at 28 and 36wkGA.

|  | **28 wkGA, PE delivery<37** | | | **36 wkGA, PE severe** | | |
| --- | --- | --- | --- | --- | --- | --- |
| **Screening result** | *All* | *High risk* | *Low risk* | *All* | *High risk* | *Low risk* |
| True positive (n) | 6 | 4 | 2 | 58 | 32 | 26 |
| False positive (n) | 13 | 9 | 4 | 508 | 126 | 382 |
| False negative (n) | 20 | 14 | 6 | 48 | 28 | 20 |
| True negative (n) | 3950 | 745 | 3205 | 3162 | 522 | 2640 |

**Table S2.** Raw data on screening for the primary outcomes using the threshold of sFlt-1:PlGF ratio of >85 at 28wkGA and >110 at 36wkGA (by maternal risk status).

|  | **28 wkGA, PE delivery<37** | **36 wkGA, PE severe** | | |
| --- | --- | --- | --- | --- |
| **Screening result** | *All* | *All* | *High risk* | *Low risk* |
| True positive (n) | 4 | 21 | 12 | 9 |
| False positive (n) | 3 | 49 | 21 | 28 |
| False negative (n) | 22 | 85 | 48 | 37 |
| True negative (n) | 3960 | 3621 | 627 | 2994 |

**Table S3.** Characteristics of composite high risk group* at 36 weeks of gestational age (n=195).

| **Maternal characteristic or birth outcome** | **High risk 36wkGA**† |
| --- | --- |
| sFlt-1:PlGF ratio >110 | 70 (36) |
| Age≥40 years | 6 (3.1) |
| Body mass index ≥35 kg/m^2^ | 25 (13) |
| Type 1 or type 2 diabetes mellitus | 5 (2.6) |
| Chronic hypertension | 56 (29) |
| Renal disease | 7 (3.6) |
| Uterine artery mean PI‡ (Doppler) highest decile | 90 (46) |
| Preeclampsia with severe features | 41 (21) |
| Preeclampsia without severe features | 43 (22) |
| Gestational hypertension | 9 (4.6) |
| Time from sampling to first onset of hypertension, weeks | 0.86 (-2.1 to 2.6) |
| Time from sampling to delivery, weeks | 3.1 (2.0 to 4.1) |
| sFlt-1:PlGF ratio by time from sampling to delivery |  |
| 1^st^ quartile (≤2.0 weeks) | 110 (61 to 172) |
| 2^nd^ quartile (2.1 to 3.1 weeks) | 95 (58 to 151) |
| 3^rd^ quartile (3.2 to 4.1 weeks) | 67 (50 to 116) |
| 4^th^ quartile (≥4.2 weeks) | 64 (46 to 85) |
| Gestational age, weeks | 39.4 (38.3 to 40.4) |
| Birth weight, g | 3140 (2820 to 3430) |
| Birth weight, centile | 33 (13 to 57) |
| Small for gestational age | 41 (21) |
| Induction of labor | 91 (47) |
| Mode of delivery |  |
| Spontaneous vaginal | 95 (49) |
| Assisted vaginal | 37 (19) |
| Intrapartum caesarean | 39 (20) |
| Pre-labor caesarean | 24 (12) |

Data are expressed as median (IQR) or n (%) as appropriate.

*Composite risk status at 36wkGA: high risk was defined as sFlt-1:PlGF ratio of >38 with maternal risk factors, or sFlt-1:PlGF ratio>110 irrespective of maternal risk factors. Time from sampling to first onset of hypertension is given for the 93 (48%) women who had preeclampsia or gestational hypertension. Among the 41 women who had severe preeclampsia, 30 (73%) had severe hypertension, 30 (73%) had proteinuria, 10 (24%) had low platelets (at median GA [IQR] = 39.4 [39.0 to 40.1] weeks), 13 (32%) had elevated ALT (at median GA [IQR] = 39.3 [38.6 to 40.0] weeks), 14 (34%) had elevated creatinine (at median GA [IQR] = 39.3 [37.6 to 40.4] weeks), one (2.4%) had pulmonary edema, and one (2.4%) had severe cerebral or visual symptoms. In 14 (34%) of 41 cases severe preeclampsia was superimposed. Sex and gestational age corrected birth weight percentiles were calculated using a population-based UK reference. Small for gestational age was defined as birth weight <10^th^ percentile. Data for the variables included in the table were 100% complete within the composite high risk group. †wkGA denotes weeks of gestational age. ‡PI denotes pulsatility index.

**Table S4.** Characteristics of low risk women* at 36 weeks of gestational age (n=2,660).

| **Maternal characteristic or birth outcome** | **Low risk 36wkGA**† |
| --- | --- |
| Age, years | 30.1 (26.8 to 33.0) |
| Body mass index, kg/m^2^ | 23.7 (21.7 to 26.6) |
| Uterine artery mean PI‡ (Doppler), Z score | -0.20 (-0.76 to 0.39) |
| Preeclampsia with severe features | 20 (0.8) |
| Preeclampsia without severe features | 20 (0.8) |
| Gestational hypertension | 47 (1.8) |
| Time from sampling to first onset of hypertension, weeks | 3.3 (1.3 to 4.4) |
| Time from sampling to delivery, weeks | 4.3 (3.3 to 5.0) |
| Gestational age, weeks | 40.6 (39.6 to 41.3) |
| Birth weight, g | 3490 (3195 to 3780) |
| Birth weight, centile | 45 (26 to 68) |
| Small for gestational age | 192 (7.2) |
| Induction of labor | 799 (30) |
| Mode of delivery |  |
| Spontaneous vaginal | 1323 (50) |
| Assisted vaginal | 681 (26) |
| Intrapartum caesarean | 453 (17) |
| Pre-labor caesarean | 196 (7.4) |
| Missing | 7 (0.3) |

Data are expressed as median (IQR) or n (%) as appropriate.

*Low risk women at 36wkGA: defined as sFlt-1:PlGF ratio of ≤38 without maternal risk factors. Time from sampling to first onset of hypertension is given for the 87 (3.3%) women who had preeclampsia or gestational hypertension. Among the 20 women who had severe preeclampsia, 12 (60%) had severe hypertension, 12 (60%) had proteinuria, 3 (15%) had low platelets (at median GA [IQR] = 41.4 [39.9 to 41.4] weeks), 5 (25%) had elevated ALT (at median GA [IQR] = 40.6 [38.9 to 41.1] weeks), 9 (45%) had elevated creatinine (at median GA [IQR] = 41.1 [39.9 to 41.4] weeks), none had pulmonary edema, and 3 (15%) had severe cerebral or visual symptoms. Sex and gestational age corrected birth weight percentiles were calculated using a population-based UK reference. Small for gestational age was defined as birth weight <10^th^ percentile. Data for the variables included in the table were 100% complete within the composite high risk group. †wkGA denotes weeks of gestational age. ‡PI denotes pulsatility index.

**Table S5.** Area under the ROC curve for the secondary outcomes and sub-types of preeclampsia.

|  | **Outcome** | **c statistic (95% CI)** |
| --- | --- | --- |
| **Measurement of sFlt-1:PlGF ratio at 28wkGA** | |  |
|  | Preeclampsia, onset of hypertension <37wkGA | 0.70 (0.65 to 0.75) |
|  | Gestational hypertension, delivery <37wkGA | 0.88 (0.85 to 0.92) |
|  | Gestational hypertension, onset of hypertension <37wkGA | 0.49 (0.38 to 0.60) |
|  | Preeclampsia or gestational hypertension, onset of hypertension <37wkGA | 0.66 (0.61 to 0.71) |
|  | De novo preeclampsia, delivery <37wkGA | 0.80 (0.68 to 0.92) |
|  | Superimposed preeclampsia, delivery <37wkGA | 0.78 (0.63 to 0.94) |
|  | Preeclampsia with renal dysfunction, delivery <37wkGA | 0.92 (N/A to 1.00) |
|  | Preeclampsia with HELLP syndrome, delivery <37wkGA | 0.83 (0.49 to 1.00) |
|  |  |  |
| **Measurement of sFlt-1:PlGF ratio at 36wkGA** | |  |
|  | Preeclampsia without severe features | 0.76 (0.72 to 0.80) |
|  | Severe gestational hypertension | 0.70 (0.59 to 0.81) |
|  | Any gestational hypertension | 0.62 (0.56 to 0.68) |
|  | Any preeclampsia or gestational hypertension | 0.76 (0.73 to 0.79) |
|  | De novo preeclampsia with severe features | 0.83 (0.77 to 0.88) |
|  | Superimposed preeclampsia with severe features | 0.78 (0.70 to 0.85) |
|  | Preeclampsia with renal dysfunction | 0.66 (0.47 to 0.84) |
|  | Preeclampsia with HELLP syndrome | 0.89 (0.81 to 0.97) |

HELLP denotes hemolysis, elevated liver enzymes and low platelets, wkGA denotes weeks of gestational age, ROC denotes receiver operating characteristic, CI denotes confidence interval.

**Table S6.** Area under the ROC curve for the analysis of preeclampsia with severe features and sFlt-1:PlGF ratio at 36wkGA, stratified by the tertile of per week increase in the sFlt1-PlGF ratio between the 28wkGA and 36wkGA measurements.

|  | **Tertile** | **Total N** | **c statistic (95% CI)** |
| --- | --- | --- | --- |
|  | 1 | 1223 | 0.54 (0.32 to 0.77) |
|  | 2 | 1222 | 0.58 (0.33 to 0.83) |
|  | 3 | 1221 | 0.66 (0.59 to 0.73) |
|  | Total | 3666 | 0.82 (0.77 to 0.86) |

In total, 3,666 women had sFlt-1 and PlGF measurements available at 28wkGA and 36wkGA. ROC denotes receiver operating characteristic, CI denotes confidence interval, wkGA denotes weeks of gestational age. P-value from the Chi-square test for the equality of the three c statistics = 0.52.

**Table S7.** Screening statistics for the primary outcomes by maternal risk status using the threshold of sFlt-1:PlGF ratio of >14.6 at 28 wkGA.

|  | **28 wkGA, PE delivery<37** | | |
| --- | --- | --- | --- |
| **Screening statistic** | *All* | *High risk* | *Low risk* |
| Sensitivity (%) | 38.5 (19.8-57.2) | 38.9 (16.4-61.4) | 37.5 (4.0-71.0) |
| Specificity (%) | 98.3 (97.9-98.7) | 96.4 (95.1-97.7) | 98.7 (98.3-99.1) |
| Positive predictive value (%) | 12.7 (5.3-20.0) | 20.6 (7.0-34.2) | 6.7 (0.0-14.0) |
| Negative predictive value (%) | 99.6 (99.4-99.8) | 98.5 (97.6-99.4) | 99.8 (99.7-100.0) |
| False positive rate (%) | 1.7 (1.3-2.1) | 3.6 (2.3-4.9) | 1.3 (0.92-1.7) |
| False negative rate (%) | 61.5 (42.8-80.2) | 61.1 (38.6-83.6) | 62.5 (29.0-96.0) |
| Positive likelihood ratio | 22.1 (12.9-37.9) | 10.9 (5.5-21.6) | 28.7 (11.2-73.6) |
| Negative likelihood ratio | 0.63 (0.46-0.85) | 0.63 (0.44-0.92) | 0.63 (0.37-1.08) |


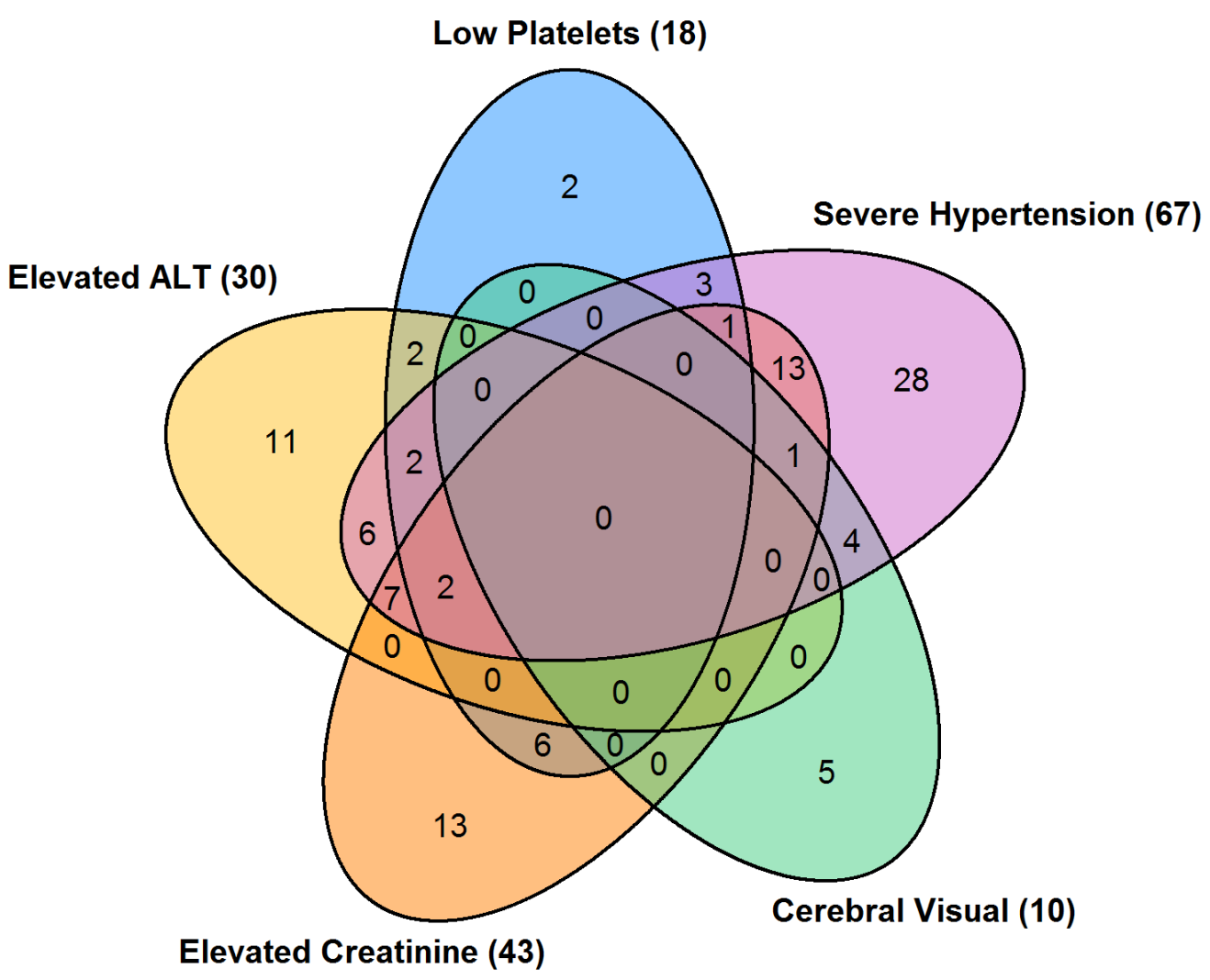


**Figure S1.** Venn diagram describing the condition of women who had pre-eclampsia with severe features (n=106).

The total number of women having each severe feature of preeclampsia is given in brackets. Cerebral Visual denotes severe cerebral or visual disturbances. Proteinuria (n=72) and pulmonary oedema (n=2) are omitted from the diagram and both of these overlap with at least one other feature.

**A**


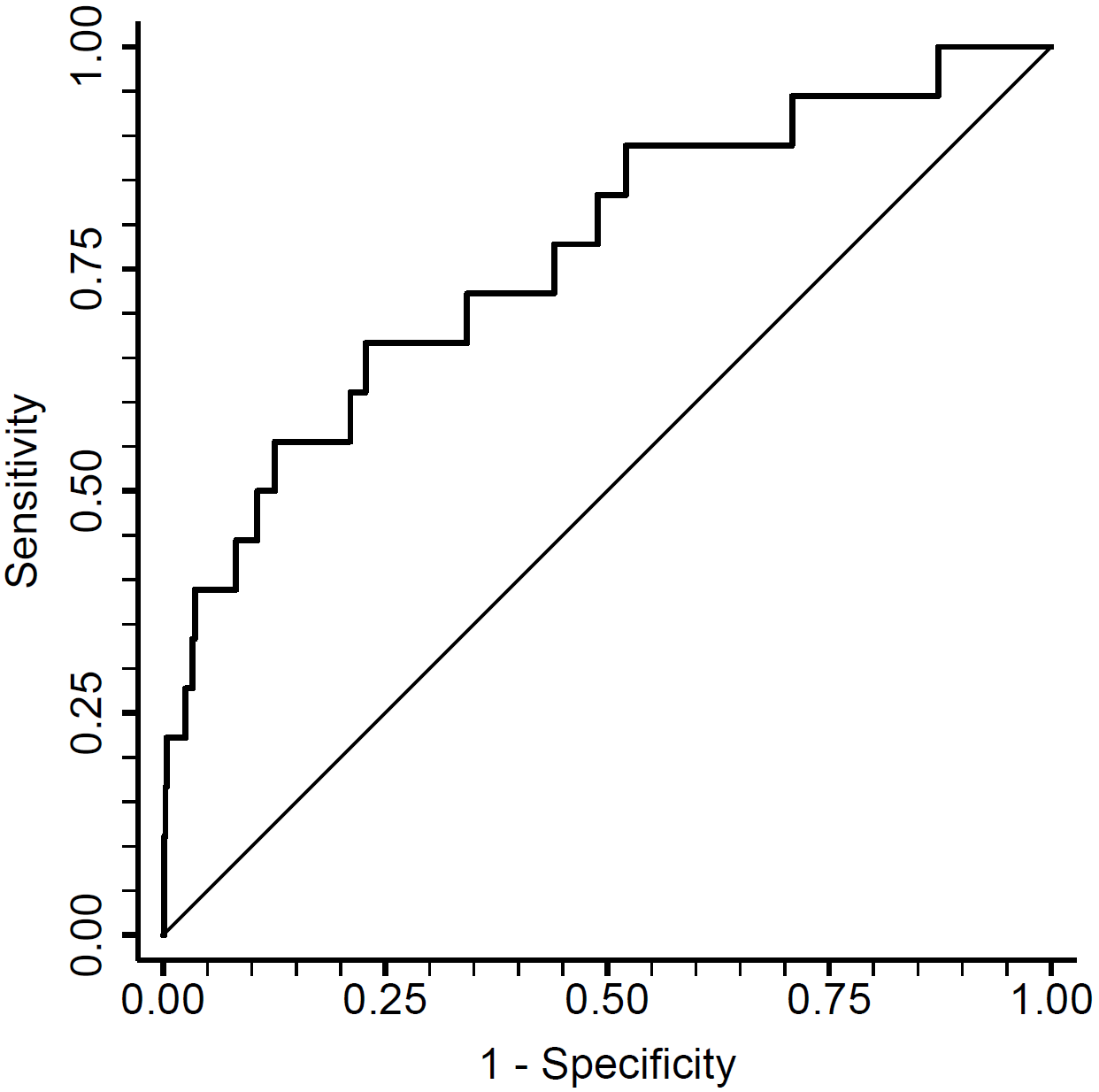


**B**


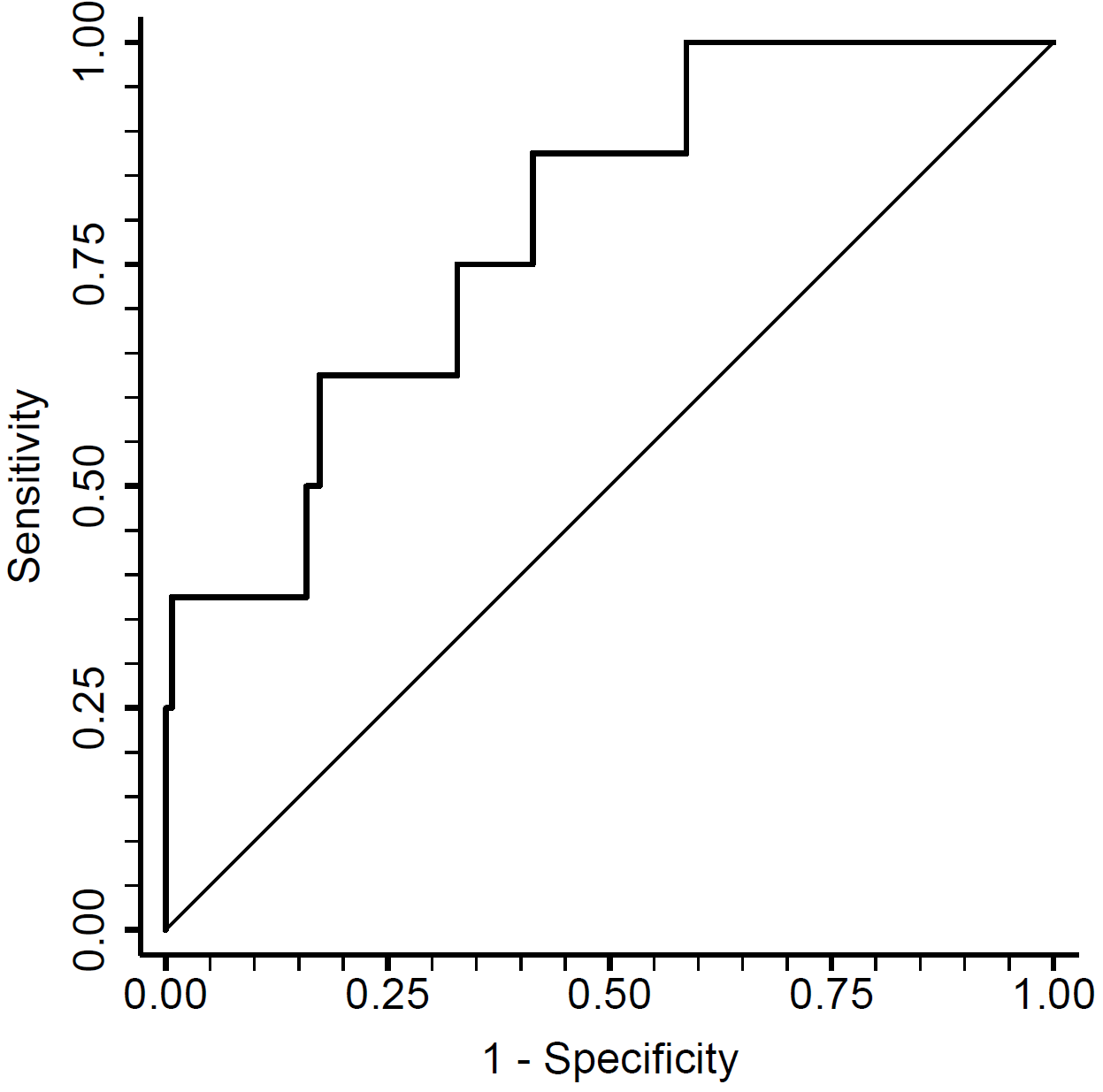


**Figure S2.** Receiver operating characteristic (ROC) curve analysis of the continuous sFlt-1:PlGF ratio at 28wkGA and primary outcome, stratified by **A.** high risk of preeclampsia, area under the ROC curve (95% CI) is 0.77 (0.64-0.89), and **B.** low risk, area under the ROC curve (95% CI) is 0.79 (0.64-0.94).

**A**


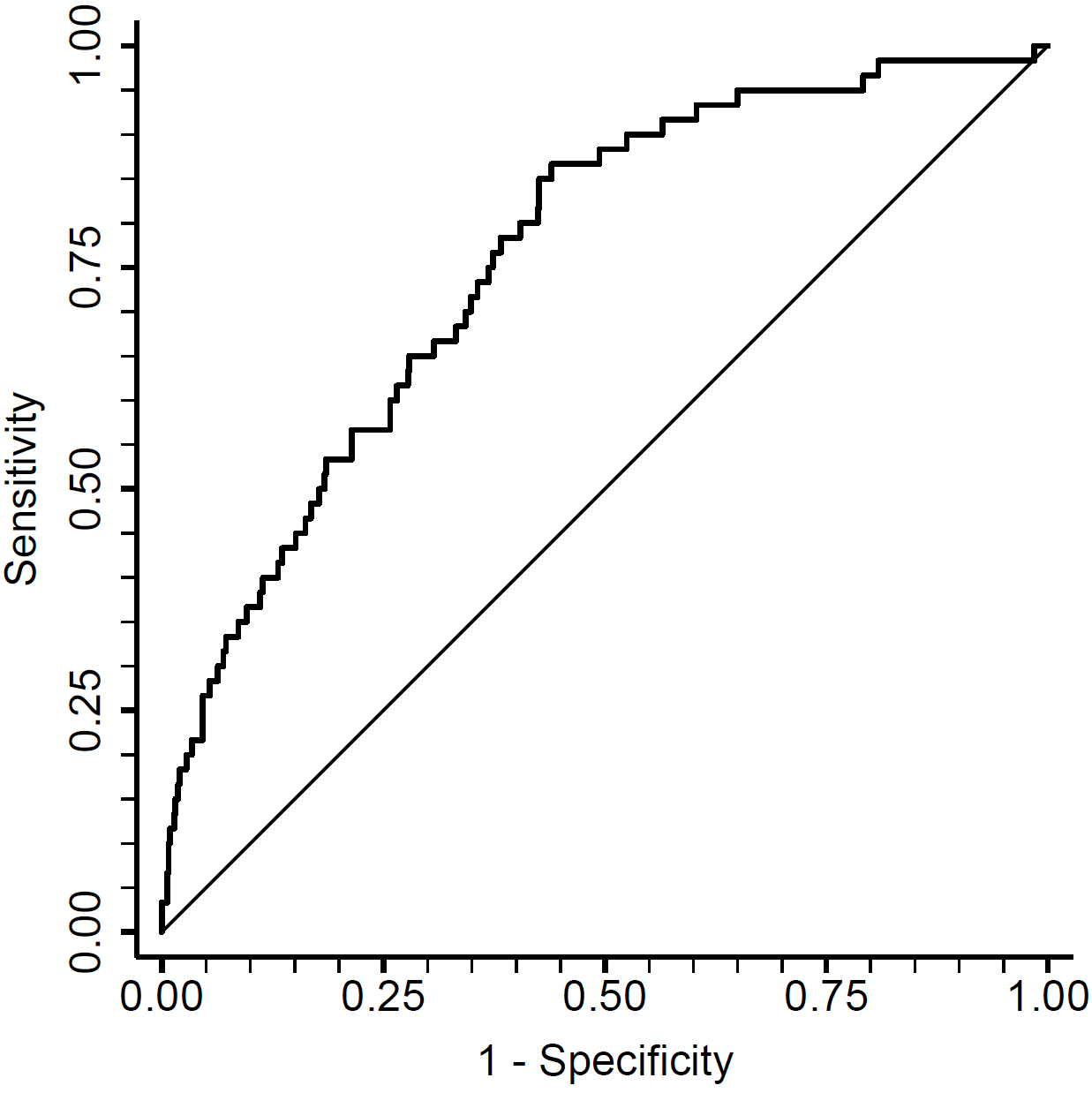


**B**


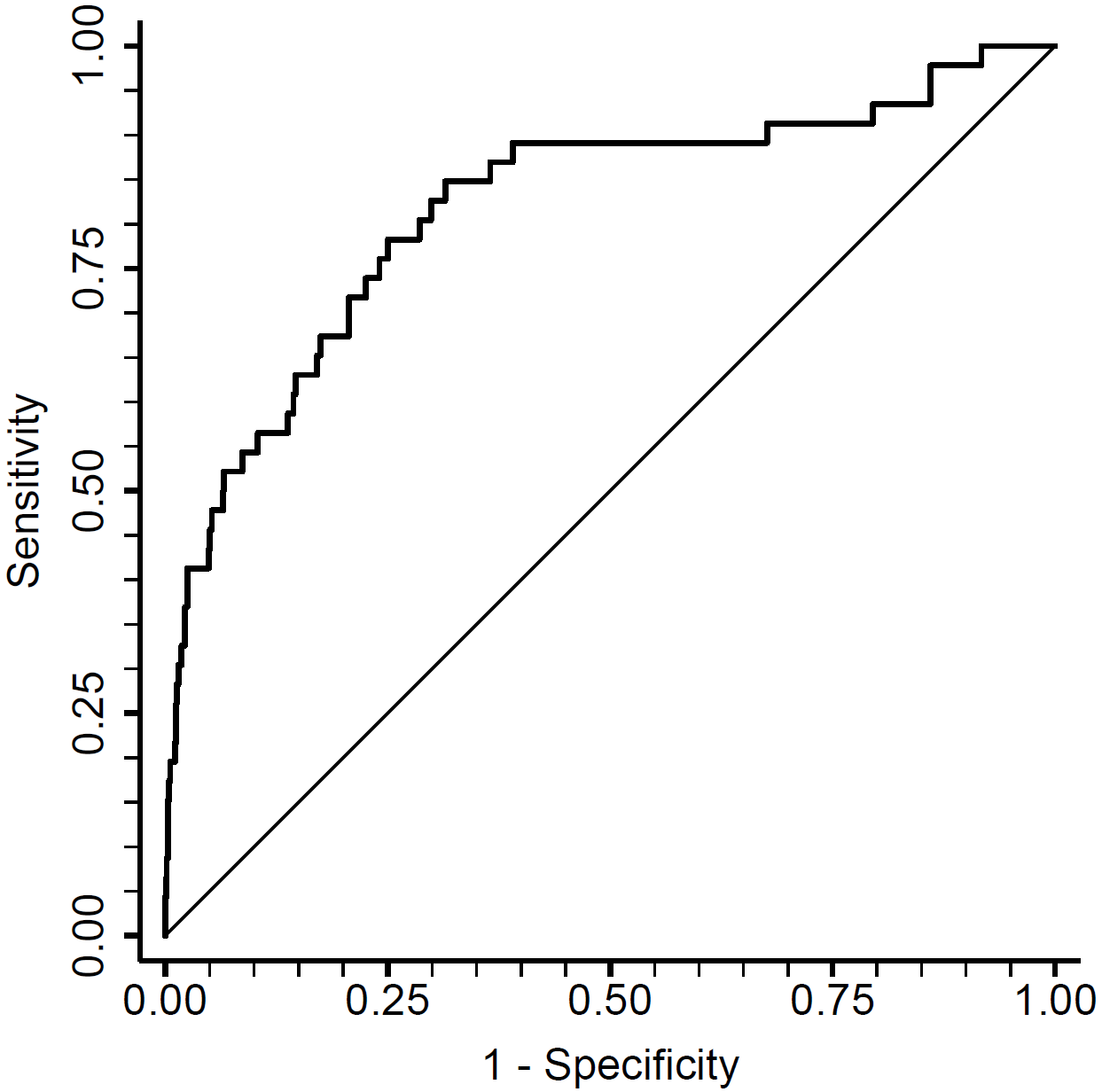


**Figure S3.** Receiver operating characteristic (ROC) curve analysis of the continuous sFlt-1:PlGF ratio at 36wkGA and primary outcome, stratified by **A.** high risk of preeclampsia, area under the ROC curve (95% CI) is 0.76 (0.70-0.82), and **B.** low risk of preeclampsia, area under the ROC curve (95% CI) is 0.82 (0.75-0.89).

**A**


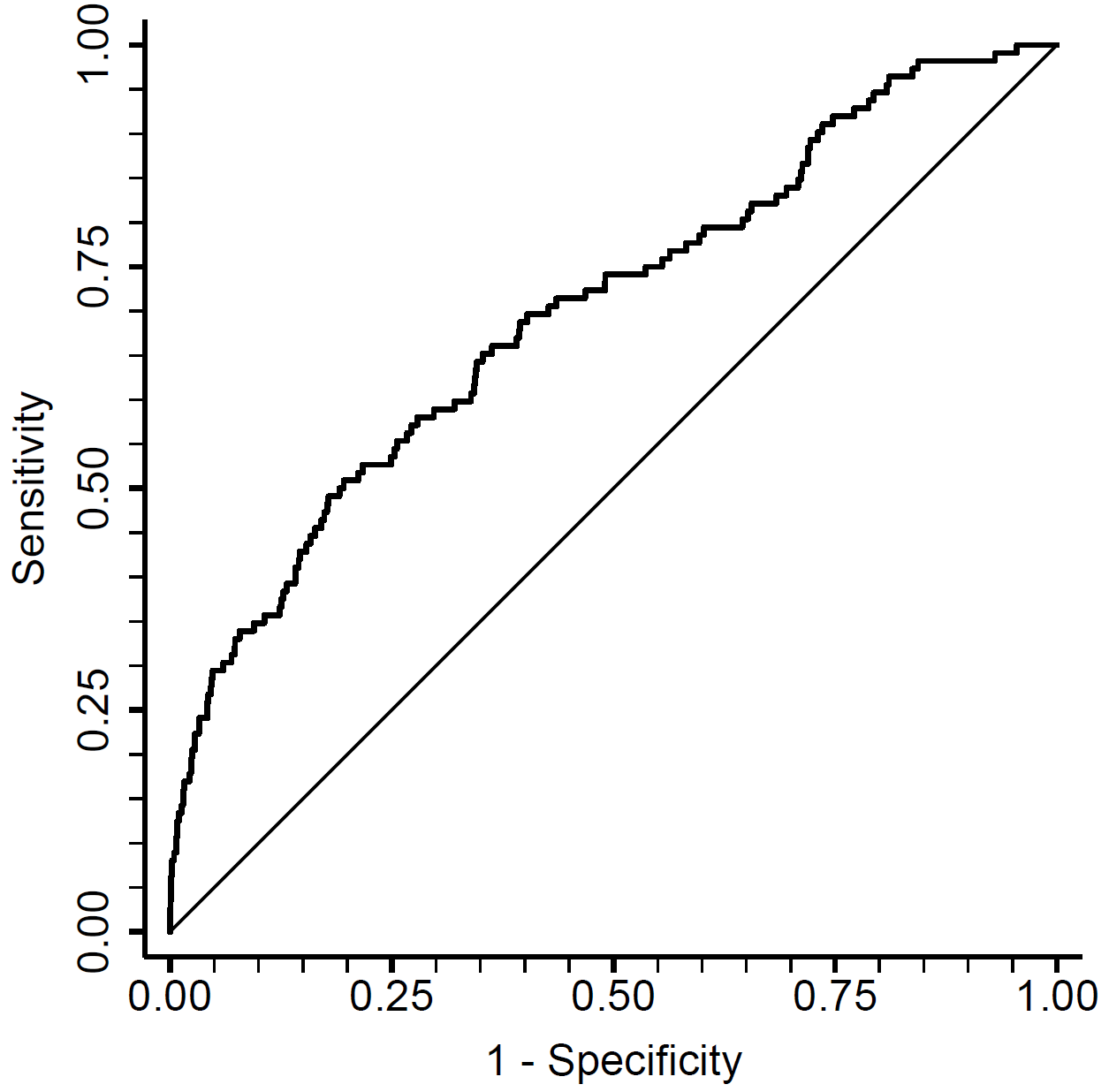


**B**


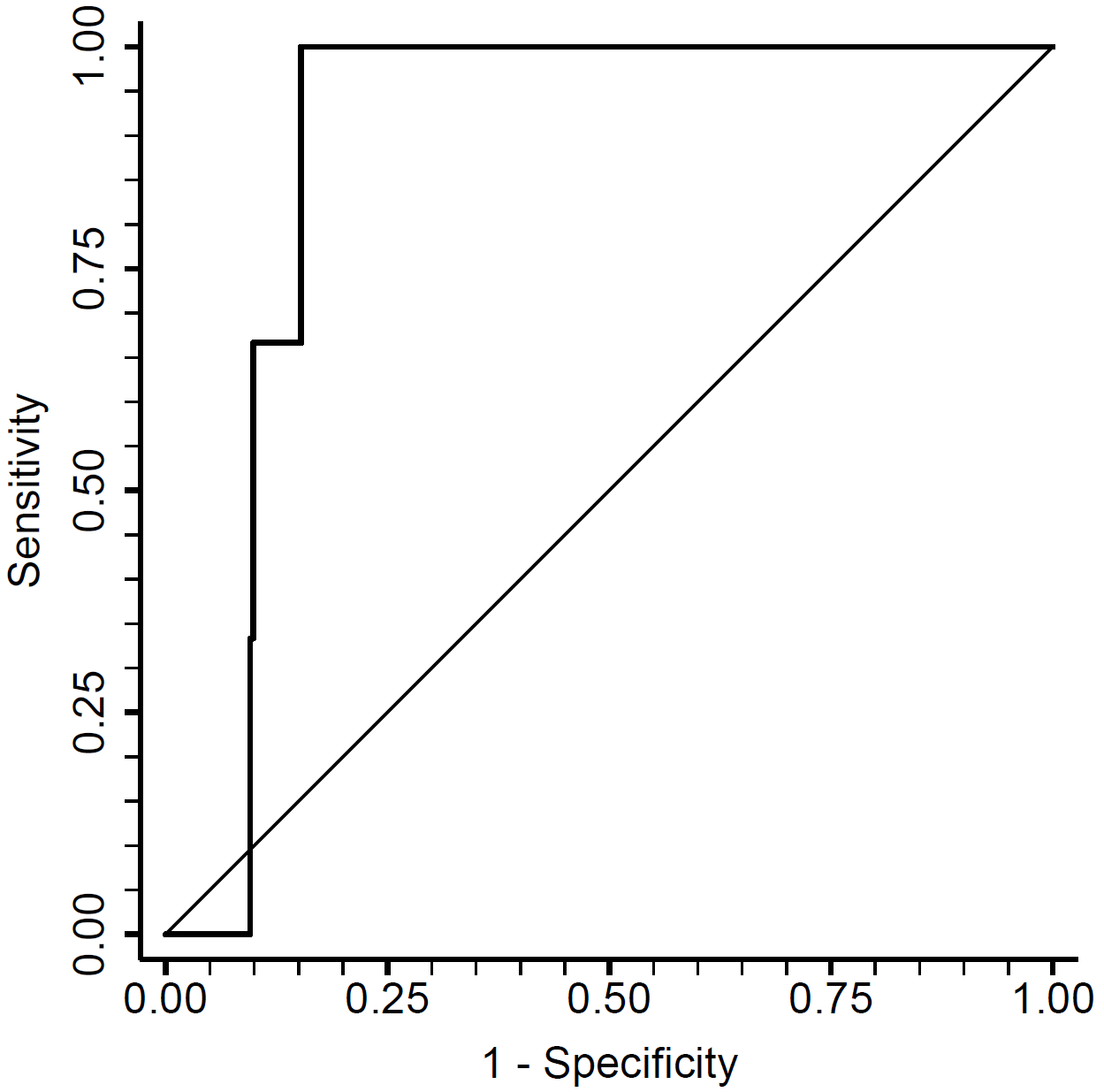


**C**


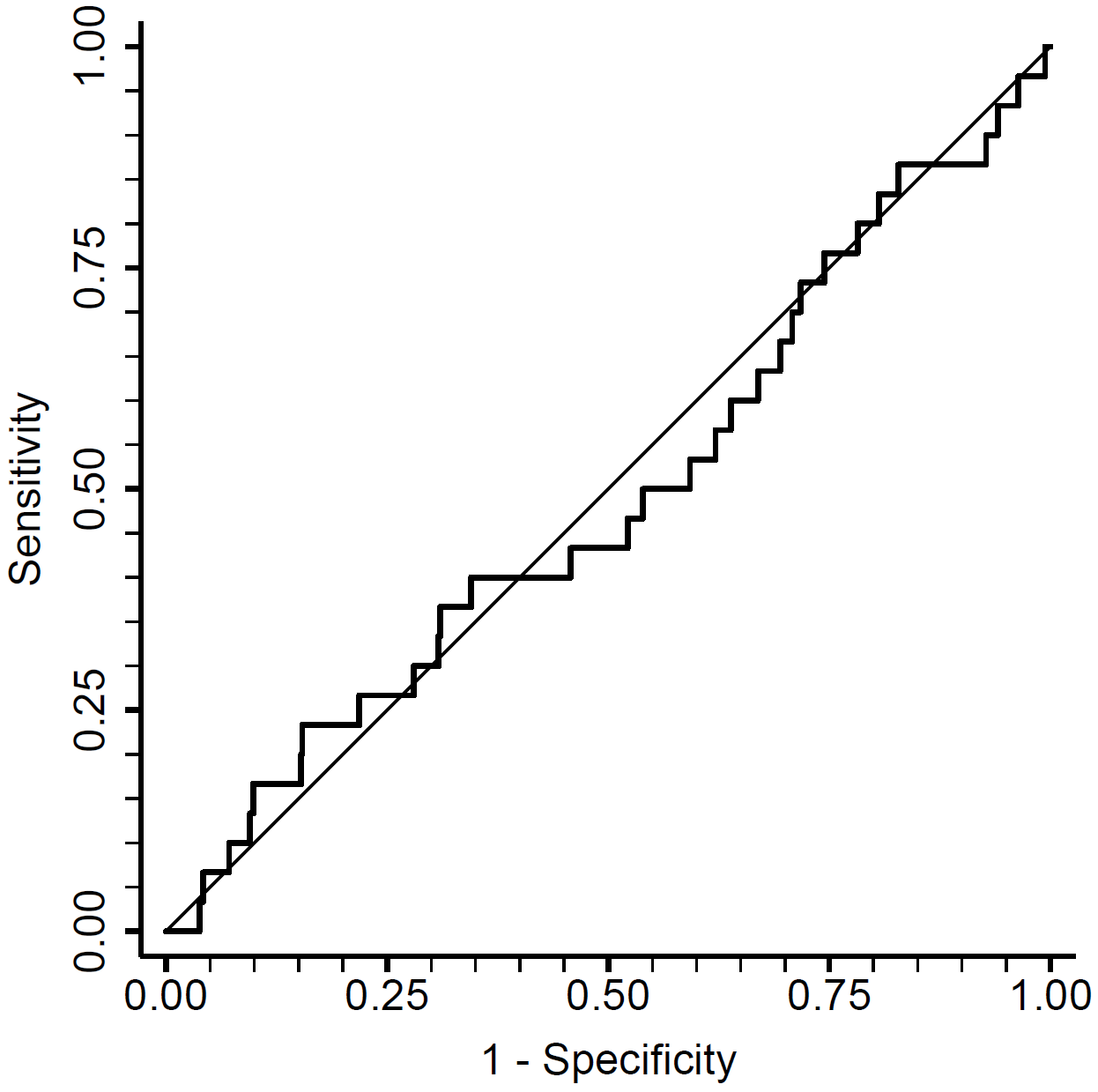


**D**


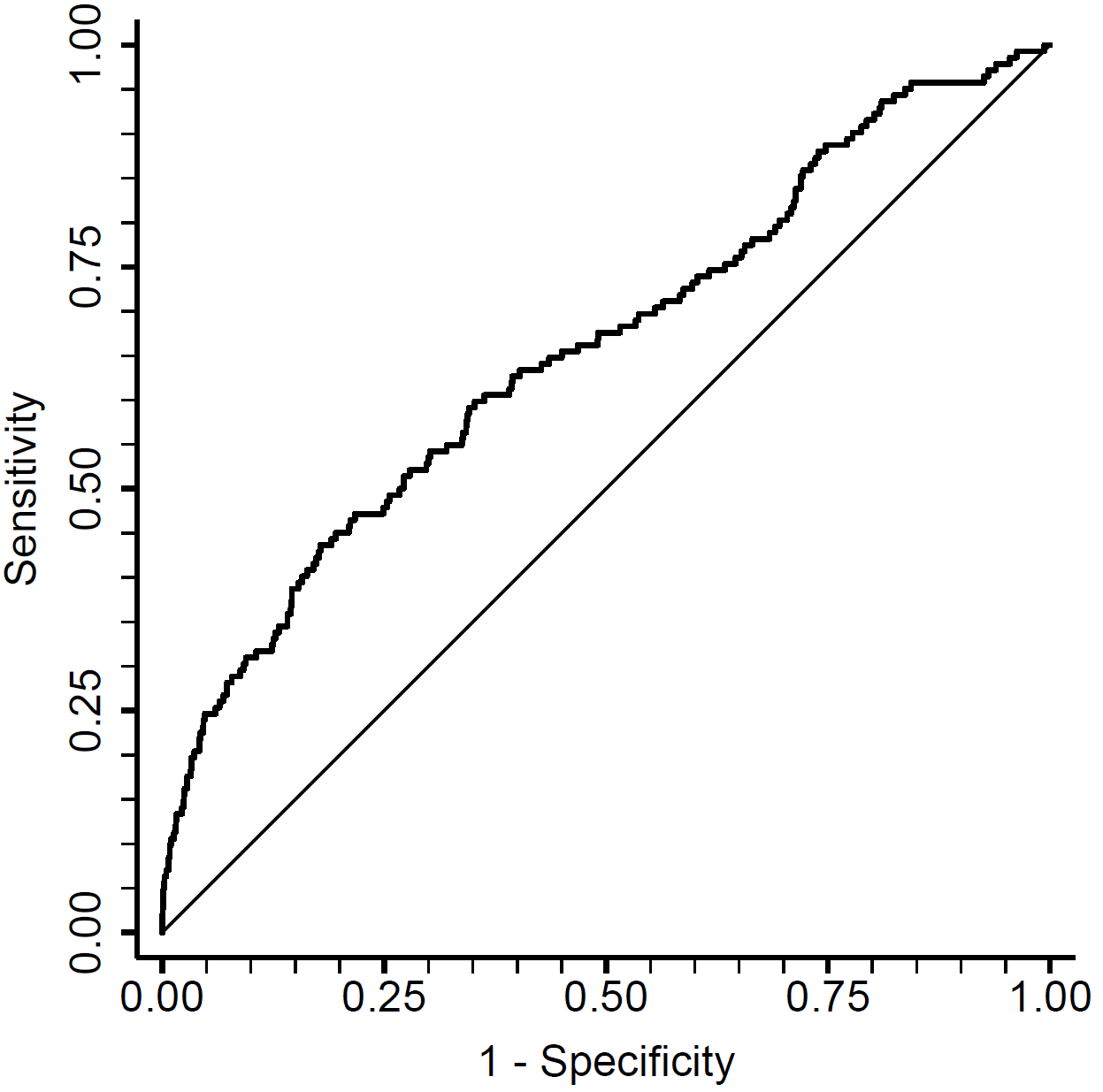


**Figure S4.** Receiver operating characteristic (ROC) curve analysis of the continuous sFlt-1:PlGF ratio at 28wkGA and secondary outcomes. Area under the ROC curve (95% CI) is given after the outcome. **A.** Preeclampsia, onset of hypertension <37wkGA, 0.70 (0.65-0.75). **B.** Gestational hypertension, delivery <37wkGA, 0.88 (0.85-0.92). **C.** Gestational hypertension, onset of hypertension <37wkGA, 0.49 (0.38-0.60). **D.** Preeclampsia or gestational hypertension, onset of hypertension <37wkGA, 0.66 (0.61-0.71).

**A**


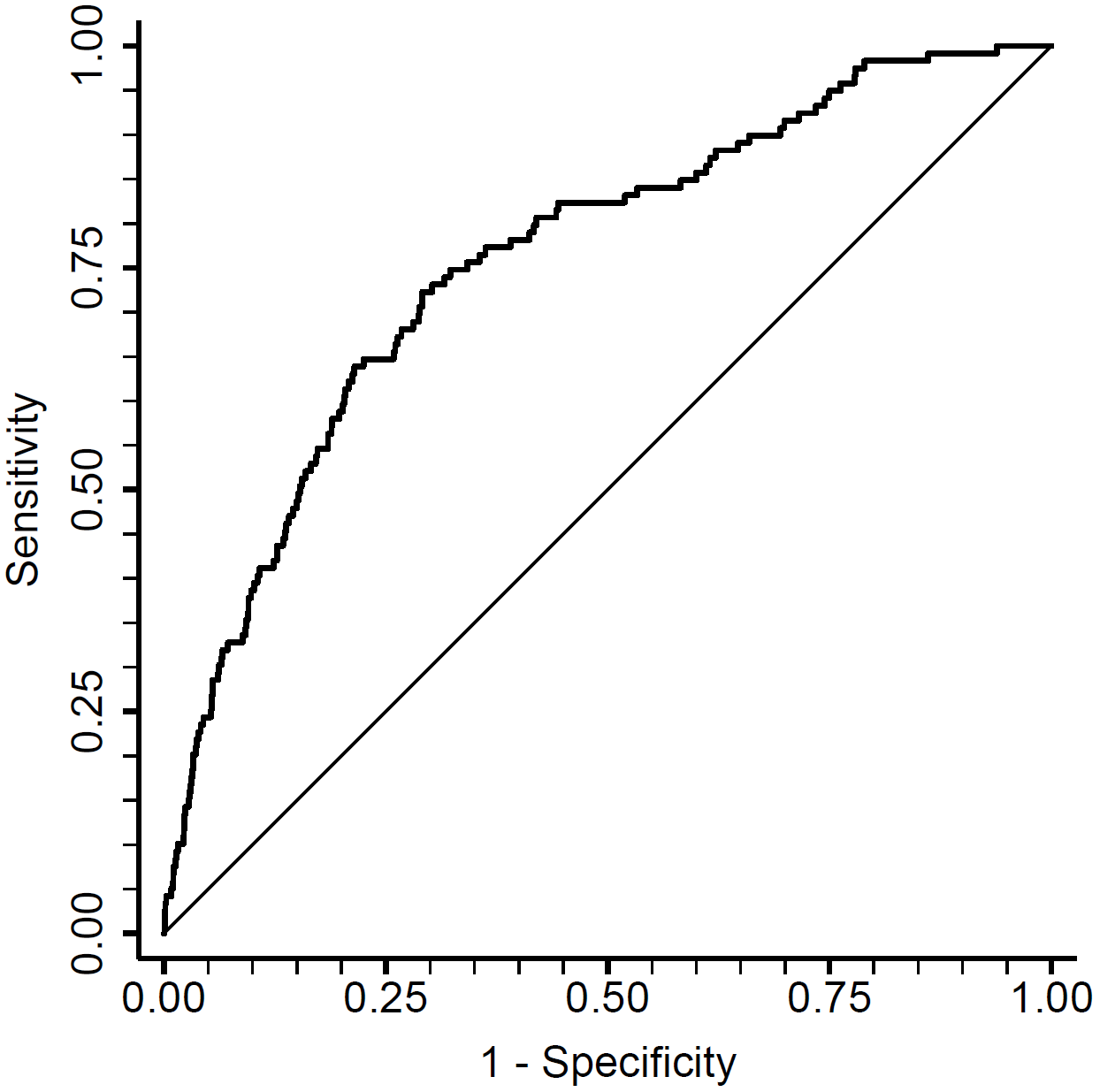


**B**


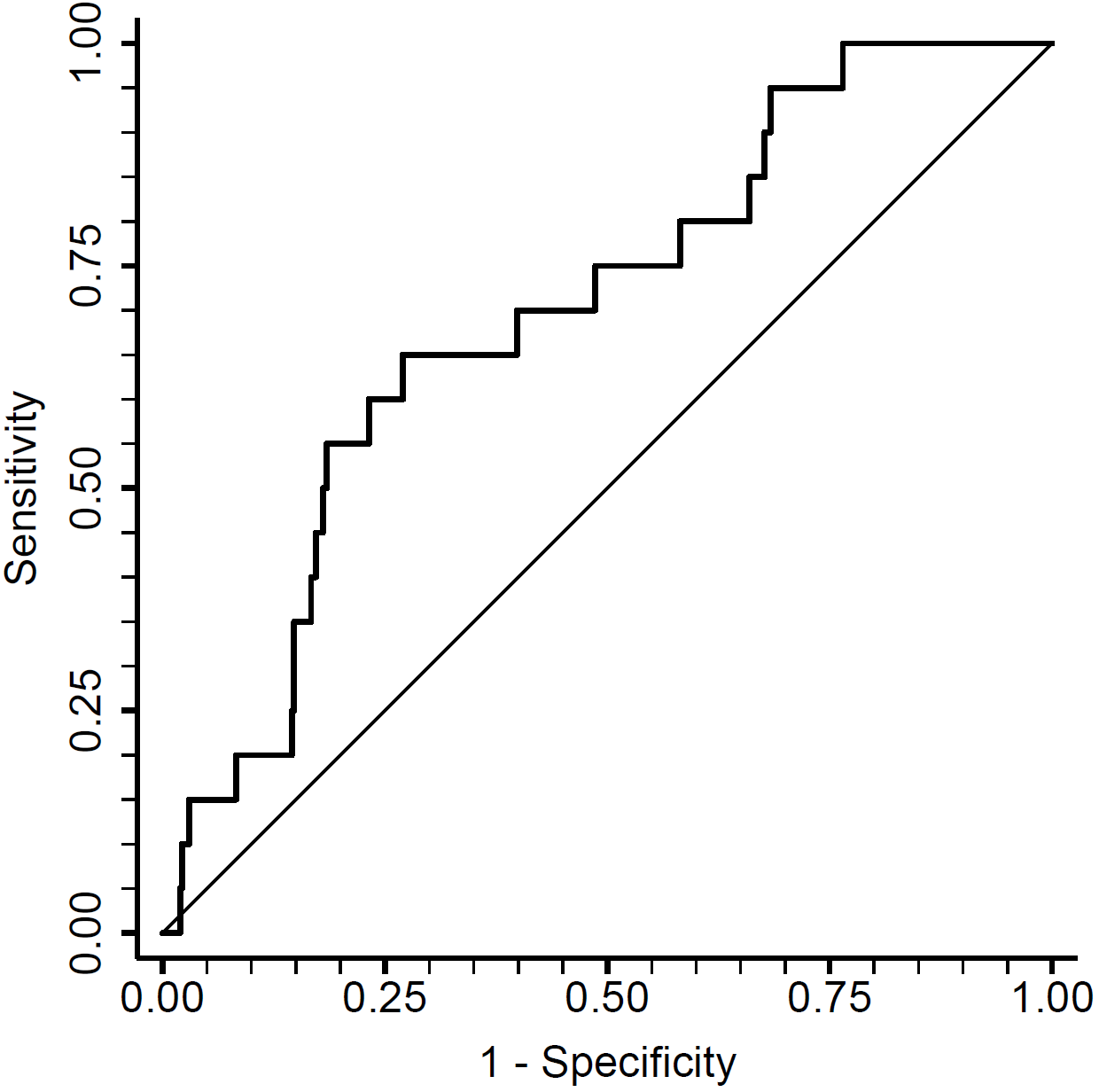


**C**


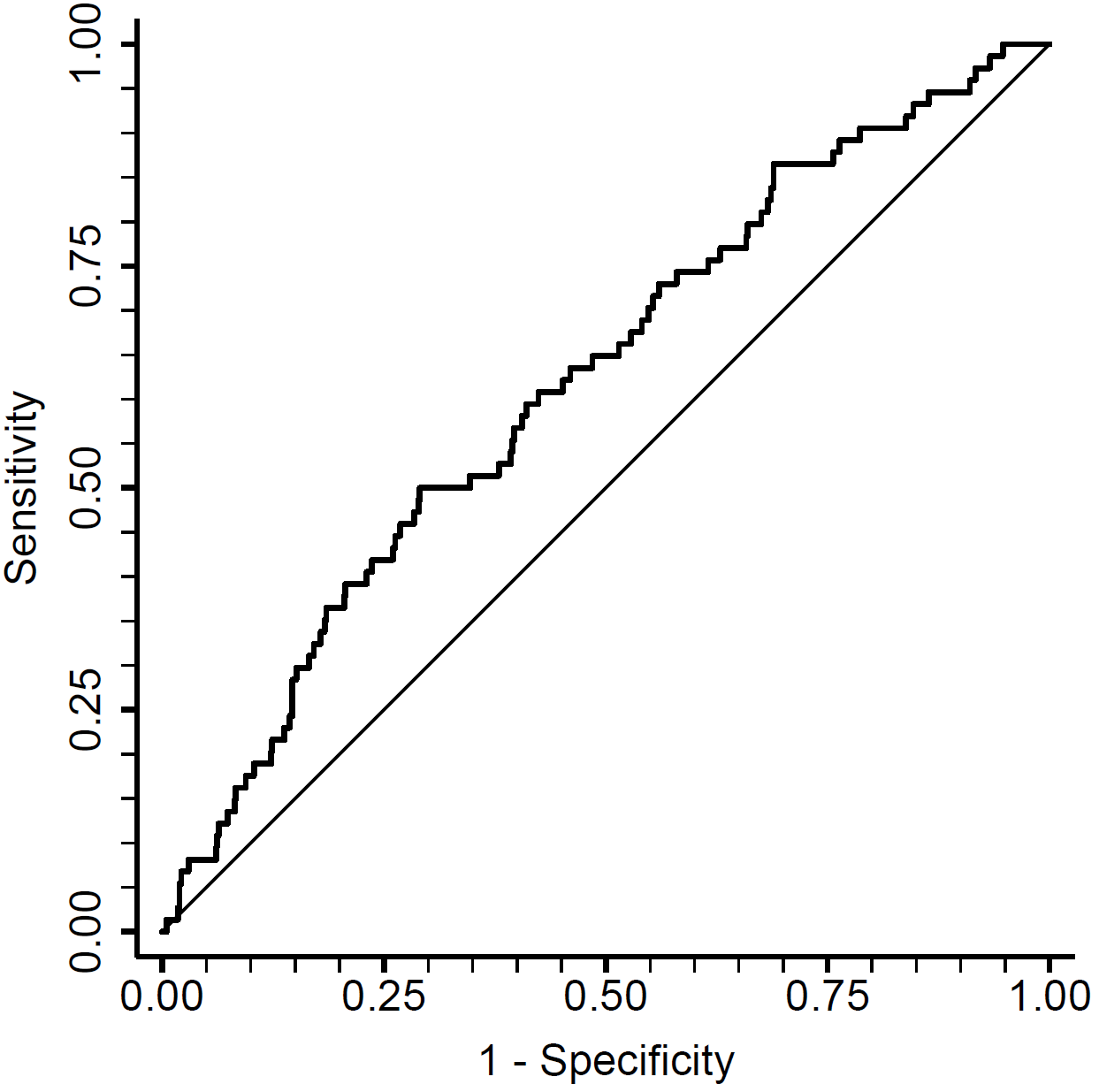


**D**


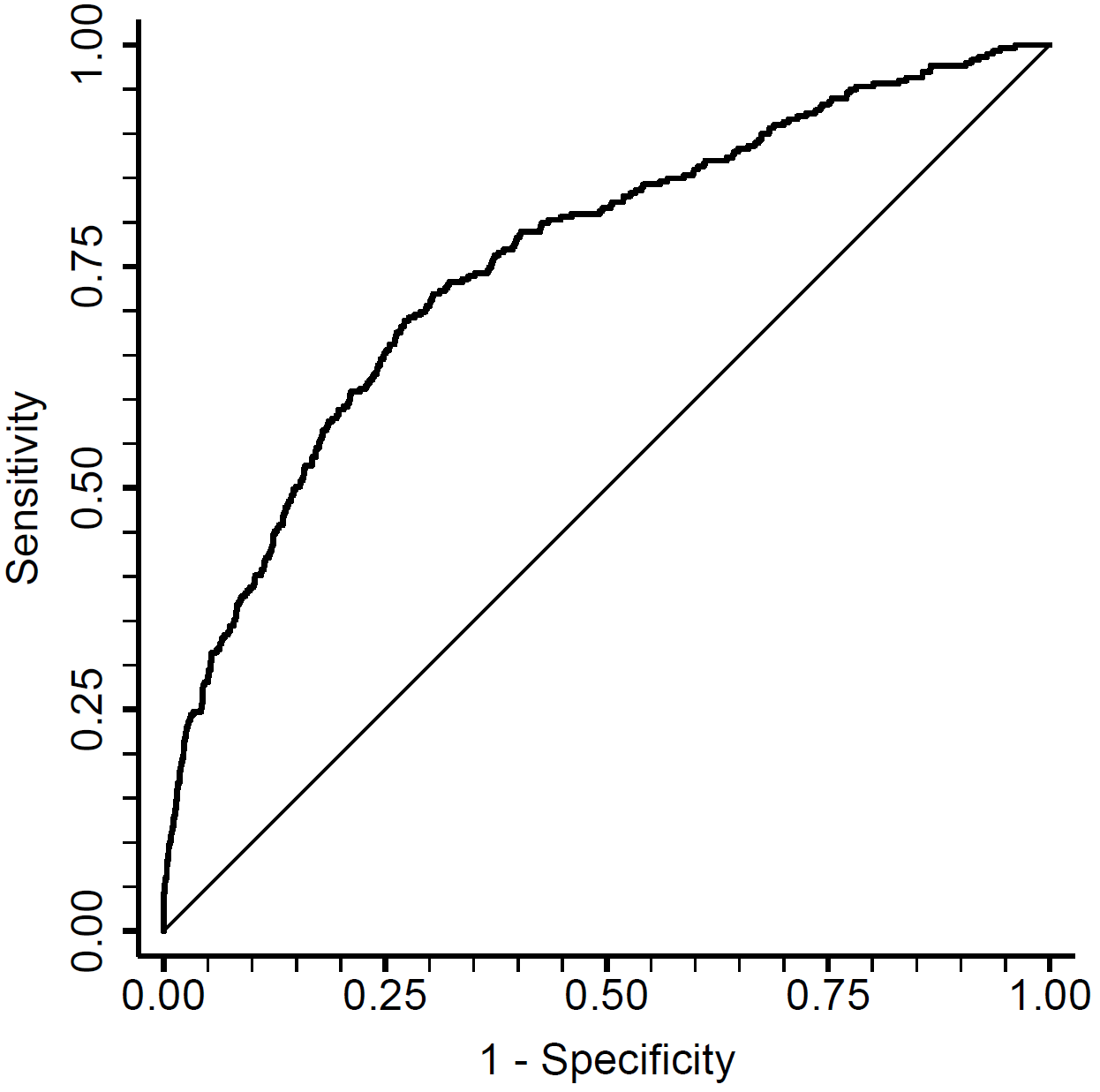


**Figure S5.** Receiver operating characteristic (ROC) curve analysis of the continuous sFlt-1:PlGF ratio at 36wkGA and secondary outcomes. Area under the ROC curve (95% CI) is given after the outcome. **A.** Preeclampsia without severe features, 0.76 (0.72-0.80). **B.** Severe gestational hypertension, 0.70 (0.59-0.81). **C.** Any gestational hypertension, 0.62 (0.56-0.68). **D.** Any preeclampsia or gestational hypertension, 0.76 (0.73-0.79).

**A**


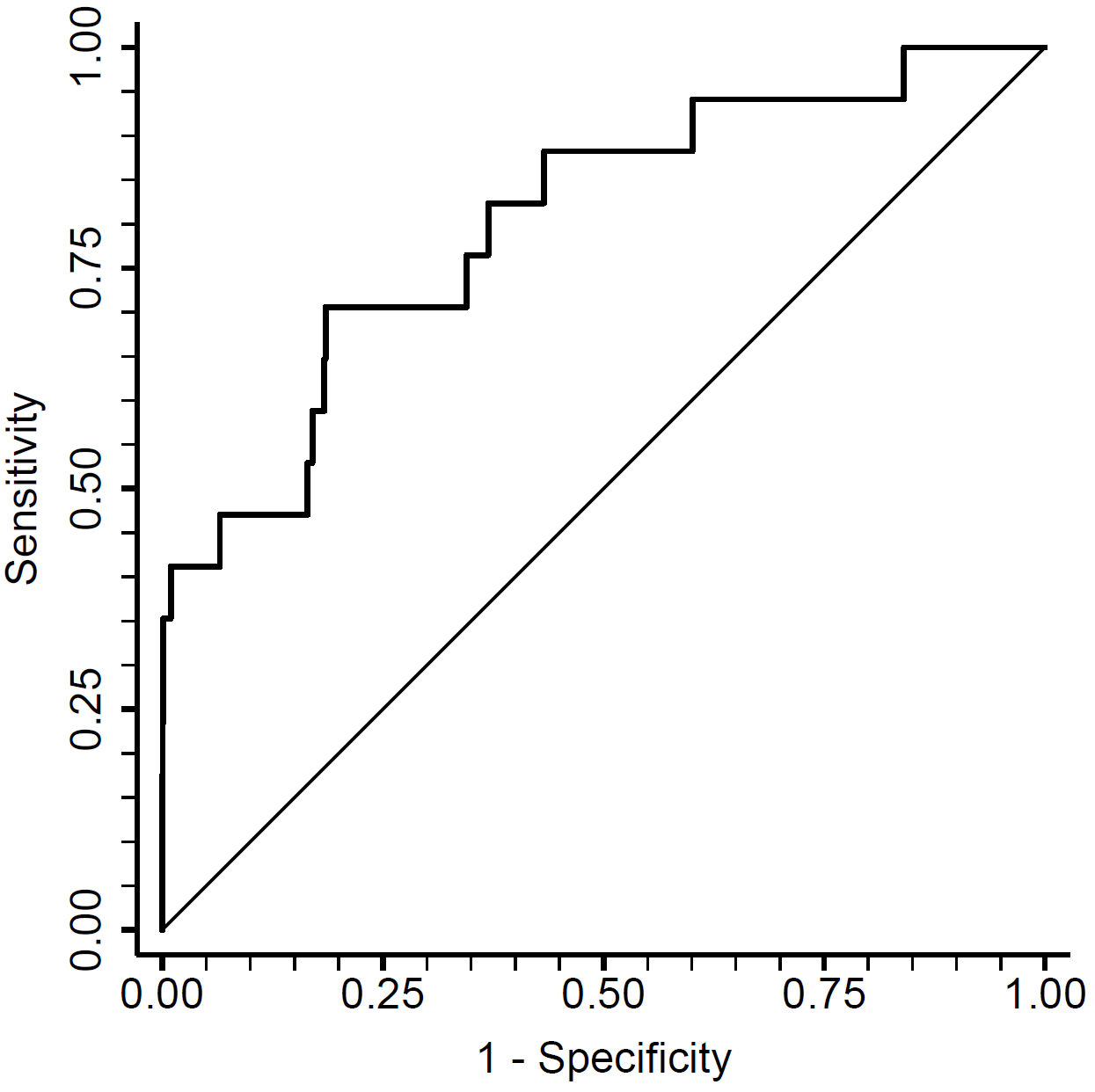


**B**


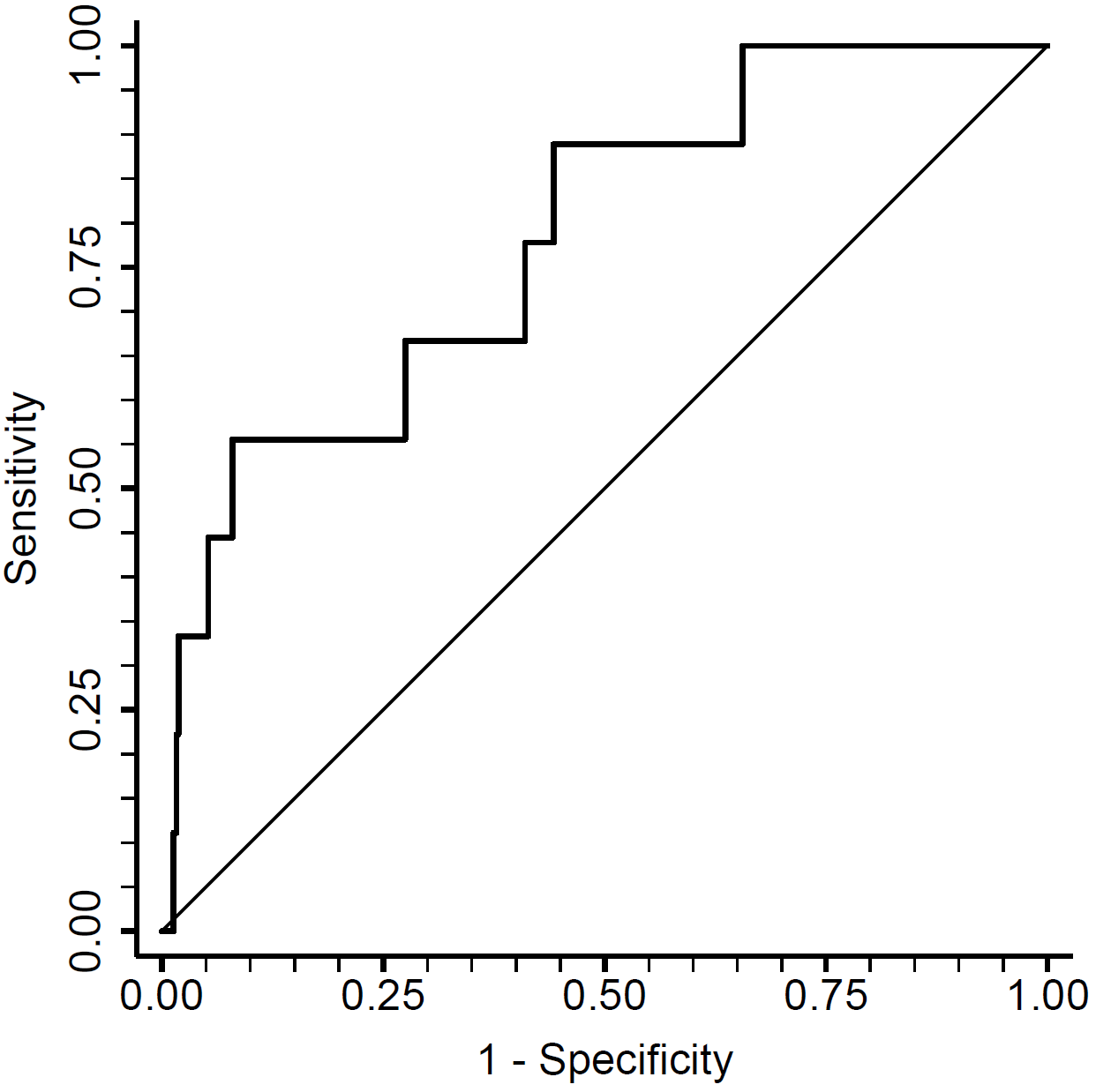


**Figure S6.** Receiver operating characteristic (ROC) curve analysis of the continuous sFlt-1:PlGF ratio at 28wkGA and **A.** de novo preeclampsia with delivery <37wkGA, area under the ROC curve (95% CI) is 0.80 (0.68-0.92), and **B.** superimposed preeclampsia with delivery <37wkGA, area under the ROC curve (95% CI) is 0.78 (0.63-0.94).

**A**


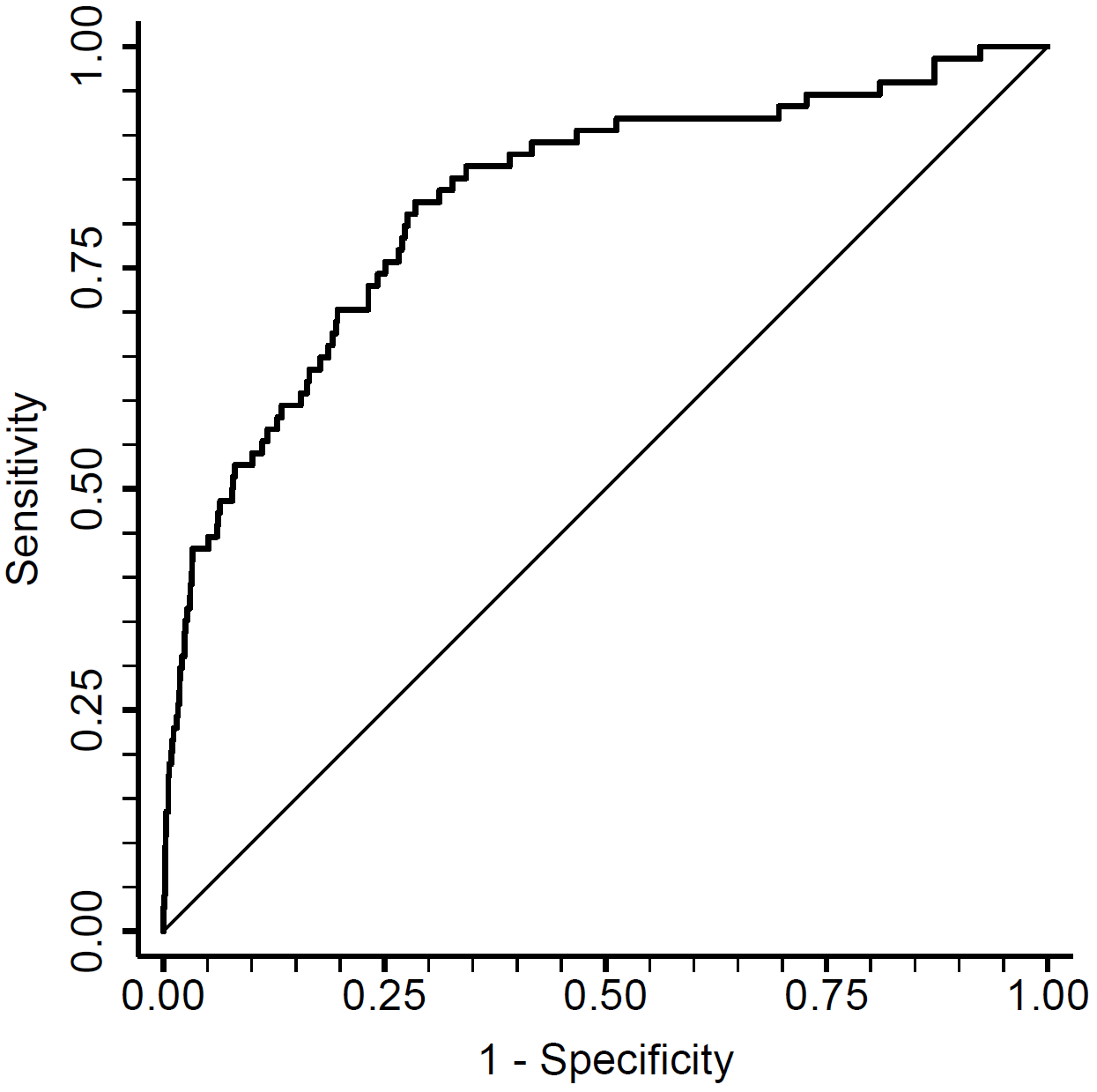


**B**


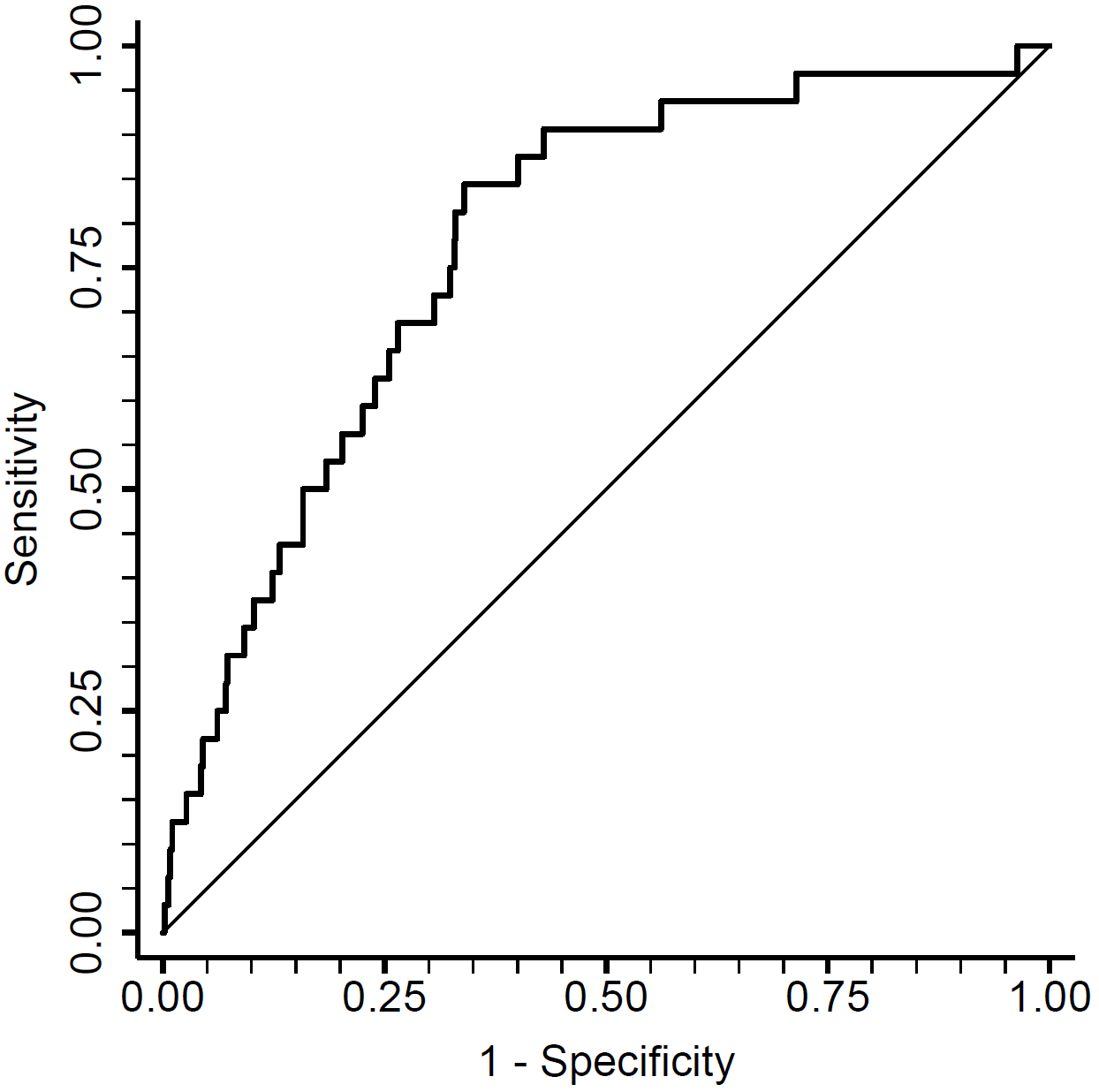


**Figure S7.** Receiver operating characteristic (ROC) curve analysis of the continuous sFlt-1:PlGF ratio at 36wkGA and **A.** de novo preeclampsia with severe features, area under the ROC curve (95% CI) is 0.83 (0.77-0.88), and **B.** superimposed preeclampsia with severe features, area under the ROC curve (95% CI) is 0.78 (0.70-0.85).

**A**


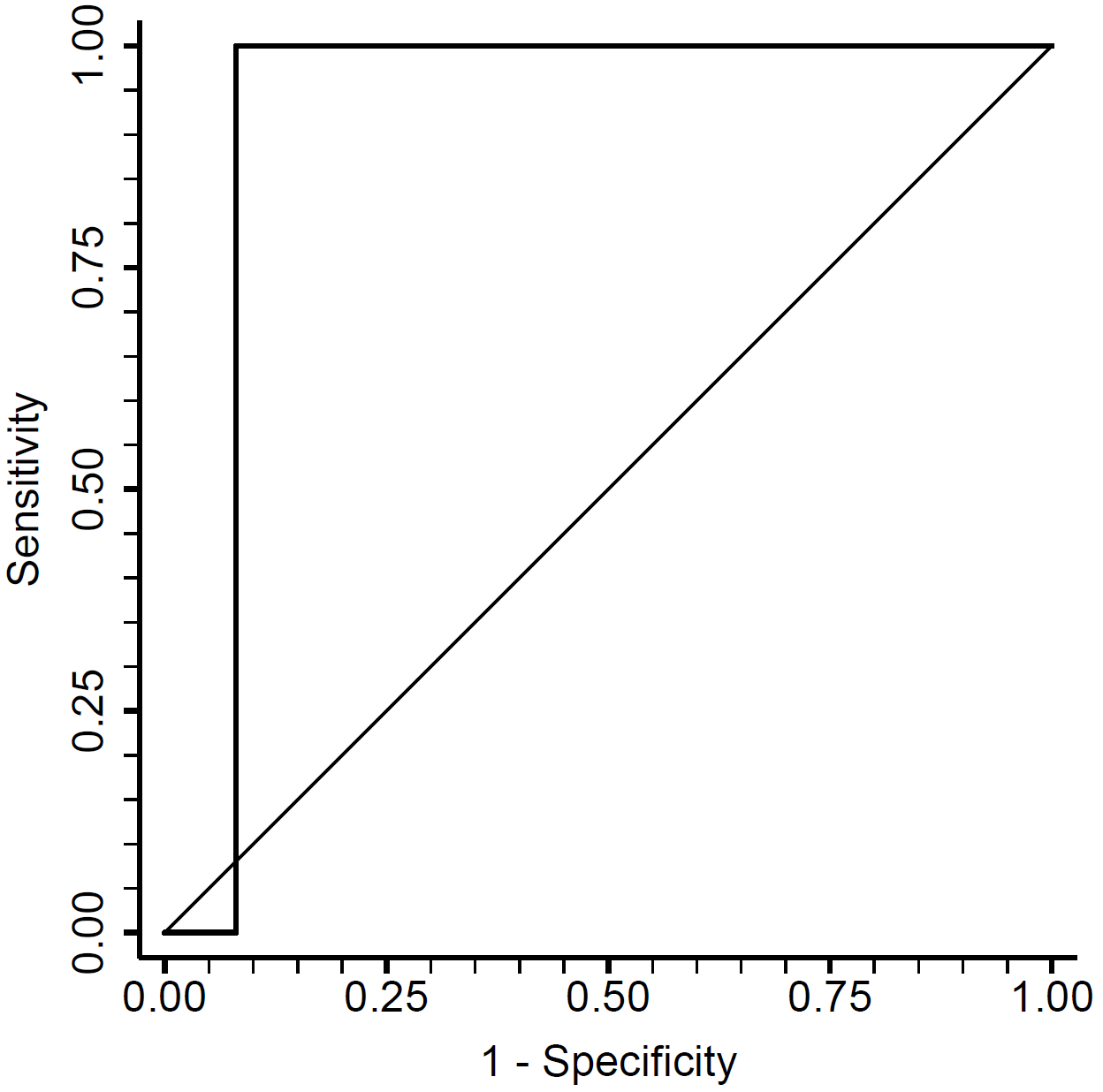


**B**


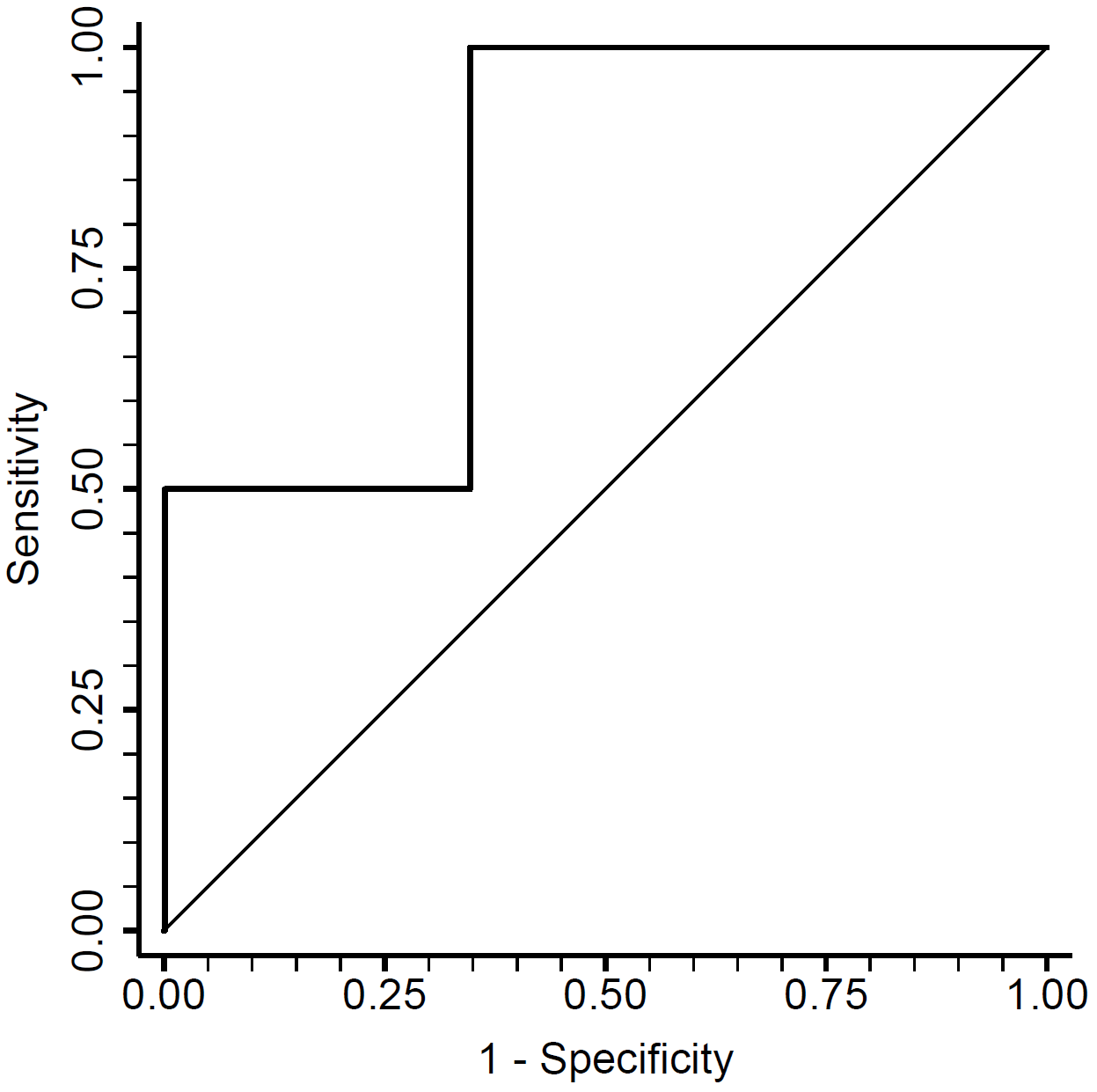


**Figure S8.** Receiver operating characteristic (ROC) curve analysis of the continuous sFlt-1:PlGF ratio at 28wkGA and primary outcome with **A.** renal dysfunction, area under the ROC curve (95% CI) is 0.92 (N/A-1.00), **B.** HELLP syndrome, 0.83 (0.49-1.00).

**A**


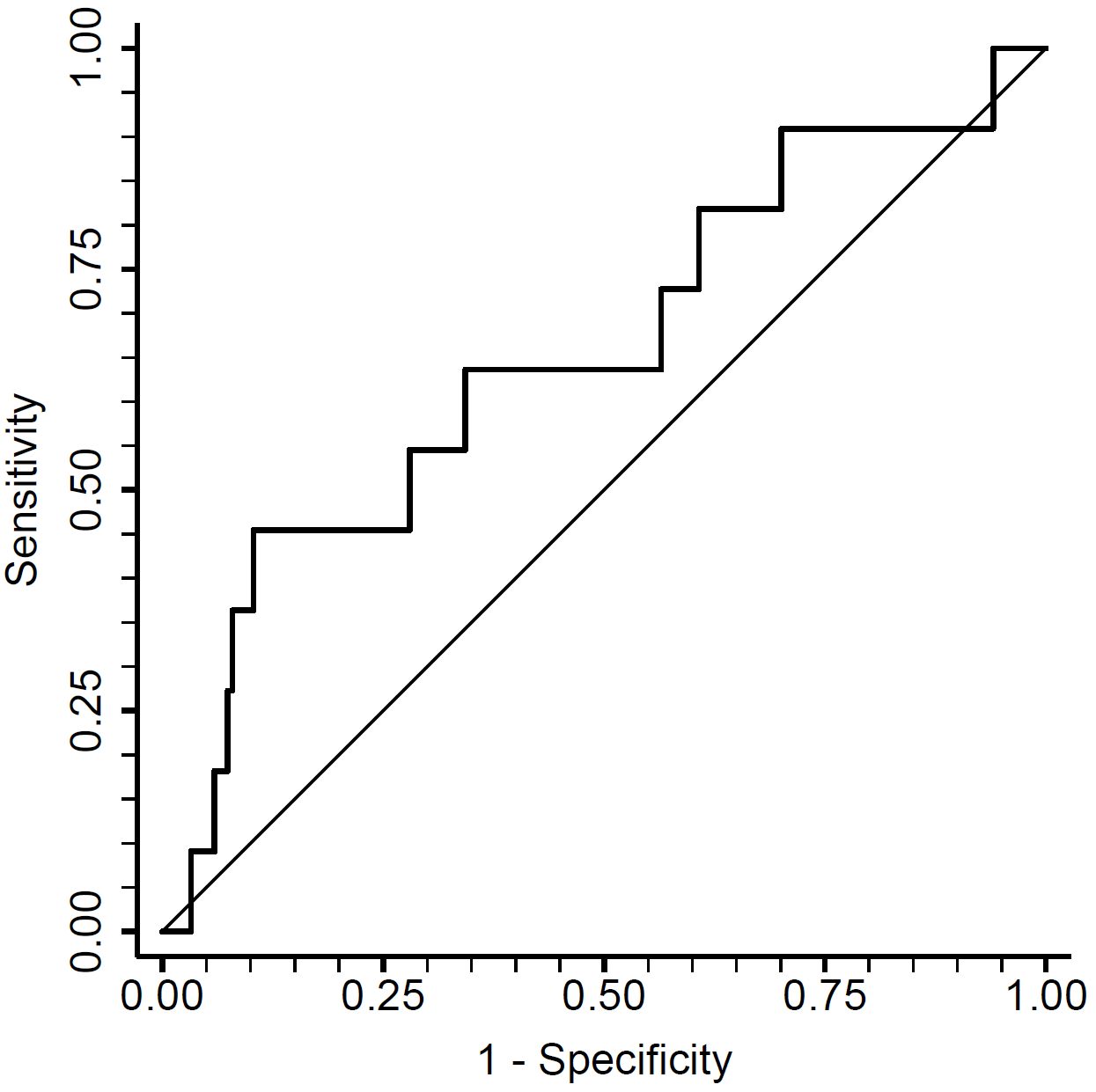


**B**


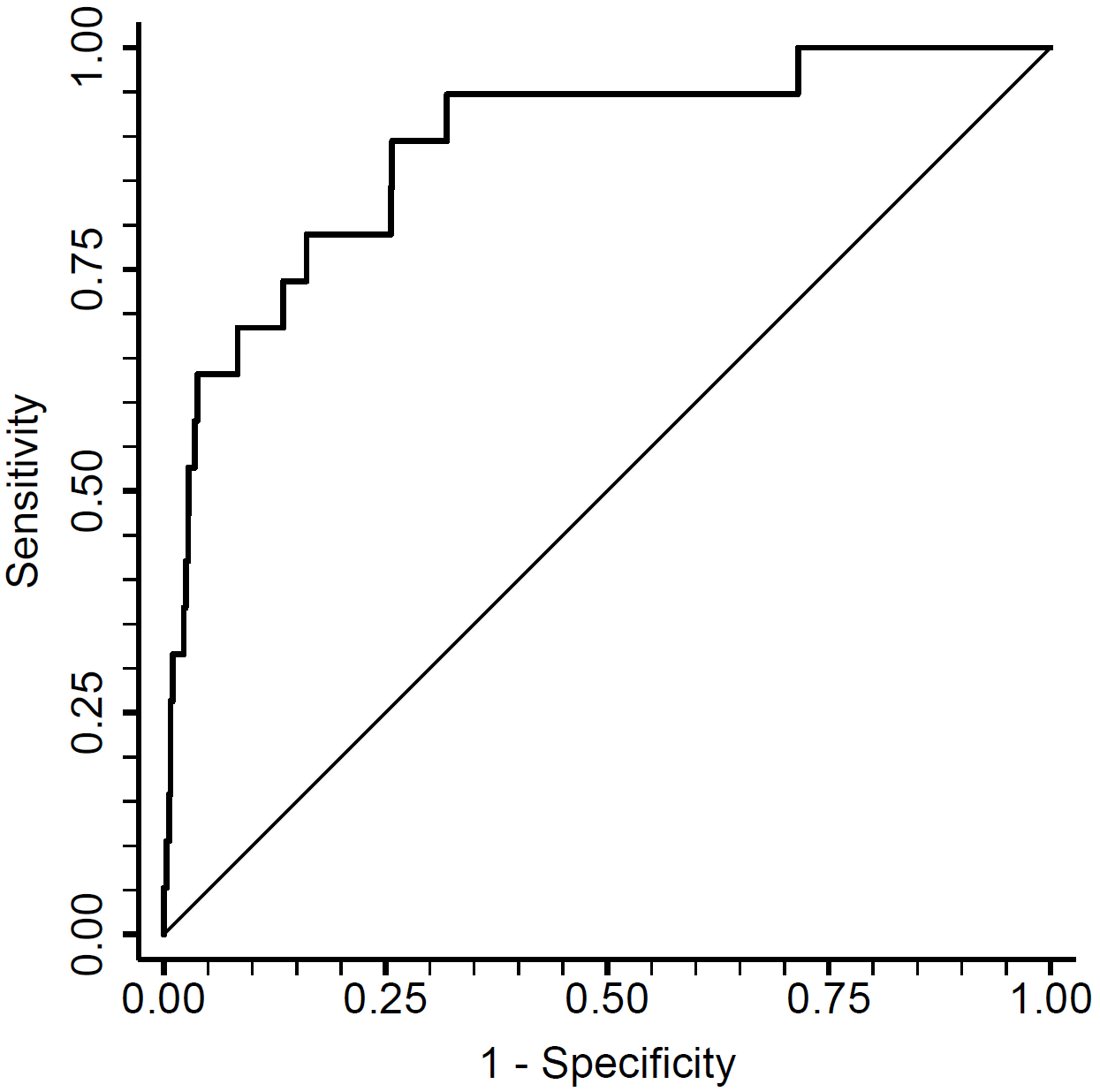


**Figure S9.** Receiver operating characteristic (ROC) curve analysis of the continuous sFlt-1:PlGF ratio at 36wkGA and preeclampsia with **A.** renal dysfunction, area under the ROC curve (95% CI) is 0.66 (0.47-0.84), **B.** HELLP syndrome, 0.89 (0.81-0.97).





**Figure S10.** Relative risk of (i) preeclampsia leading to preterm birth and (ii) preeclampsia with severe features, comparing women in the top decile of sFlt1:PlGF ratio against all other women. Cut-off points for the top decile of sFlt1:PlGF ratio are 13.30 at 20wkGA, 6.90 at 28wkGA and 49.82 at 36wkGA. The 36wkGA measurement was not analyzed in relation to preterm preeclampsia.
